# Interfacial Chemistry Limits the Stability of Deep Blue Perovskite LEDs Revealed by Operando Characterization

**DOI:** 10.1021/acsenergylett.5c00989

**Published:** 2025-06-28

**Authors:** Alessandro J. Mirabelli, Birgit Kammlander, Yang Lu, Rahul Mahavir Varma, Qichun Gu, Karen Radetzky, Thomas A. Selby, Tianjun Liu, Stefania Riva, Zimu Wei, Tien-Lin Lee, Jonathan Rawle, Håkan Rensmo, Miguel Anaya, Ute B. Cappel, Samuel D. Stranks

**Affiliations:** † Department of Chemical Engineering and Biotechnology, University of Cambridge, Philippa Fawcett Drive, Cambridge CB3 0AS, U.K.; ‡ Cavendish Laboratory, University of Cambridge, JJ Thomson Avenue, Cambridge CB3 0HE, U.K.; § Division of X-ray Photon Science, Department of Physics and Astronomy, Uppsala University, Box 516, 751 20 Uppsala, Sweden; ∥ Wallenberg Initiative Materials Science for Sustainability, Department of Physics and Astronomy, Uppsala University, 751 20 Uppsala, Sweden; ⊥ Diamond Light Source, Harwell Science and Innovation Campus, Didcot OX11 0DE, U.K.; ∇ Departamento de Fisica de la Materia Condensada, Instituto de Ciencia de Materiales de Sevilla, Universidad de Sevilla-CSIC, Avenida Reina Mercedes SN, Sevilla 41012, Spain

## Abstract

To commercialize lead halide perovskites as light-emitting diodes (LEDs), the operational device lifetime needs to be drastically improved. For this to be achieved, an understanding of degradation behavior under bias is crucial. Herein, we perform operando measurements of the structural, chemical, and electronic changes using synchrotron-based grazing-incidence wide-angle X-ray scattering and hard X-ray photoelectron spectroscopy on full-stack deep blue mixed bromide/chloride lead halide perovskite LEDs. While a clear drop in optoelectronic performance is recorded under electrical bias, the accompanying X-ray scattering data reveals only minor changes in structural properties. However, photoelectron spectroscopy reveals substantial chemical changes at the electron-injecting interface after bias is applied, including the formation of unwanted metallic lead and a new chlorine species that is not in the perovskite structure. These operando approaches give important structural and interfacial perspectives to reveal the degradation mechanisms in these LEDs and highlight the need to address the top electron-injecting interface to realize step-changes in operational stability.

## Graphical abstract

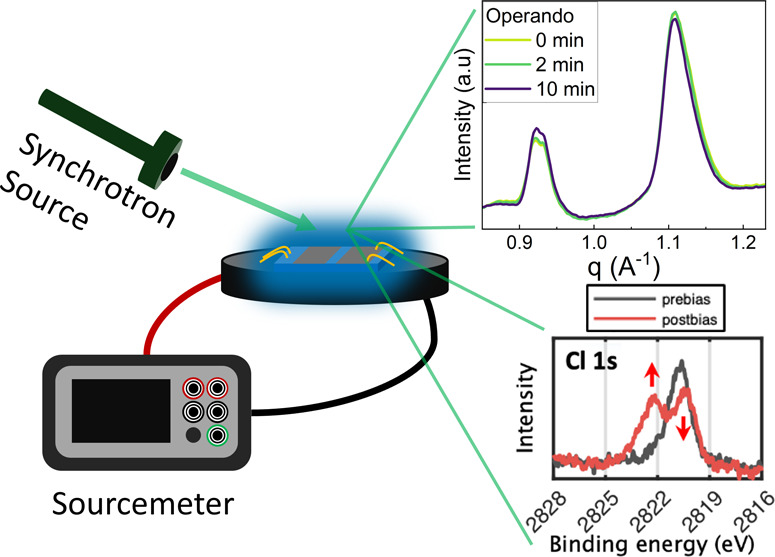

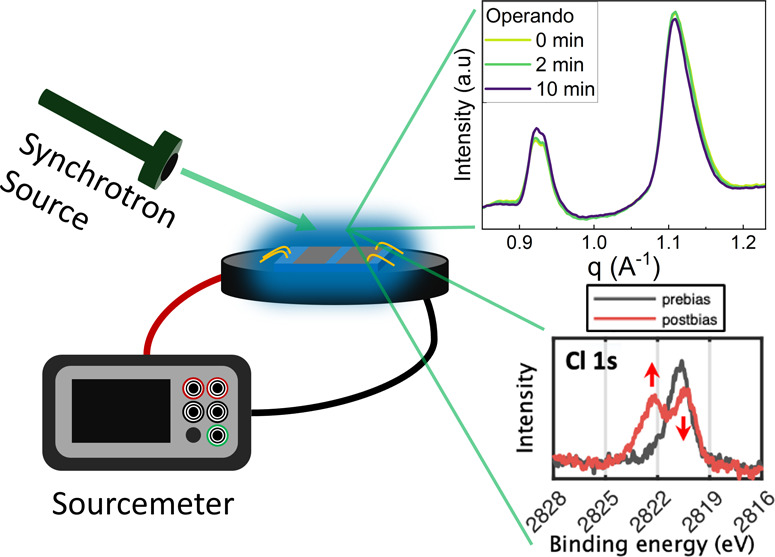

Lead halide perovskites are promising for optoelectronic applications such as light-emitting diodes (LEDs), solar cells, and photodetectors due to their favorable and tunable properties.^1,2^ For perovskite LEDs (PeLEDs), different strategies have been implemented to boost their performance over the past decade, with external quantum efficiencies (EQEs) now exceeding 25%.^3^ While properties such as performance and luminance have improved drastically, operational stability and device longevity are still far from sufficient.^4^ As the degradation pathways are currently not well understood, insights into the underlying mechanisms will be critical to their mitigation and the eventual commercialization of lead halide PeLEDs. State-of-the-art blue PeLEDs have reached efficiencies close to their red and green counterparts, over 20% for sky blue (490 nm) and 10% for deep blue (465 nm) emission.^5^ However, out of the three primary colors, blue emitters are the most unstable, as their operational lifetime remains the shortest, with T_50_ (time taken to reach 50% of the initial luminance value) of, at best, only a few hours for sky blue devices and even shorter for deep blue.^6^ A wider blue range can be achieved by either mixing chloride and bromide halides or confining the emitter by incorporating organic spacer molecules to introduce low-dimensional perovskite structures.^7–9^ Mixed-halide devices have high luminance values but are particularly susceptible to phase segregation under operation, leading to bromide- and chloride-rich regions. This is reflected in an emission red shift over time due to charge carriers funneling from wide-bandgap to newly formed narrow-bandgap regions.^10^ While adopting single-halide perovskites could mitigate segregation issues, the introduction of organic spacers to the formula adds new instability elements, such as poor interactions between crystal planes and charge injection.^11^ A blend between bulk 3D and low-dimensional perovskite ensures that the system retains good energy transfer properties while also benefiting from efficient emissive exitonic character.^12–14^

While much research has been focused on investigating the degradation of lead halide perovskite films,^15–18^ there is limited knowledge concerning full devices. This is comprehensible, as the greater number of layers and interfaces involved complicates the interpretation of many measurements, especially when the devices are operated. Recent reports have revealed that, in mixed bromide/chloride-based blue PeLEDs, chloride ions in particular drift under the influence of an electric field and chloride oxidation plays a significant role in device failure.^19^ In particular, ion migration is reported to be the underlying mechanism responsible for operational instability, as charges accumulate at the interfaces and ease the charge injection due to a lowered effective injection barrier.^20^ While a reversible phenomenon is possible when only mild degradation occurs, this is not the case for prolonged device operation.^21^ These findings prove more severe with increasing chloride content to achieve deeper blue emission, as this lowers the migration energy barrier of the system, and the resulting PeLEDs possess T_50_ lifetimes of only a few minutes.^22^ We recently tracked the operando photoluminescence (PL) and electroluminescence (EL) of sky blue PeLEDs over time, revealing the formation of chloride- and bromide-rich regions at defect sites.^23^ However, the precise location and type of chemical and structural changes within the stacks are not understood.

Grazing-incidence wide-angle X-ray scattering (GIWAXS) is a powerful technique for relaying crystallographic information on both the surface and bulk of thin-film samples. This characterization technique has become increasingly popular for halide perovskites, especially those in lower dimensional structures (e.g., 2D or quasi-2D) where directionality and preferential alignment properties are important to resolve.^24^ Its surface-sensitive and depth-dependent character has been employed to understand how 2D and 3D perovskite phases can arrange optimally to boost both performance and stability in perovskite films.^25,26^ By adding the operando aspect, our intention is to correlate degradation under different stressing conditions in full devices, bringing the technique closer to real-world conditions.^27–29^ In recent work, we reported halide remixing in the out-of-plane direction in mixed-cation, mixed-halide perovskite solar cells during operation.^30^ Hard X-ray photoelectron spectroscopy (HAXPES) is a more surface-sensitive technique that gives electronic and compositional information on materials, as well as insights into band bending and (interfacial) energy alignment.^31^ Band alignment can be studied directly on structures with thin top layers, while core-level spectra give information on compositional and chemical changes.^31^ We previously investigated full solar cell stacks via operando HAXPES to follow the photovoltage throughout the different device layers, where substantial photovoltage was generated at the gold contact/hole transport layer interface.^32^

Overall, chemical and structural changes under operation are not yet understood. Here, we seek to fill this gap in understanding of deep blue LEDs by simultaneously measuring structural, chemical, and electronic device degradation through operando GIWAXS and HAXPES measurements on full-stack deep blue PeLEDs. The concurrent device data shows signs of optoelectronic degradation and failure, yet from diffraction data we find that the perovskite crystal structure remains mostly unaltered during LED operation, where only a small fraction undergoes a phase transition from orthorhombic to trigonal. However, we find substantial interfacial chemical changes after applying bias in our HAXPES measurements. The appearance of a new chloride species together with the formation of metallic lead were recorded after our PeLEDs were operated for an extended period. These interfacial changes then became sites for nonradiative recombination, causing eventual device failure, which is reflected in the luminance and current drop during operation. This work demonstrates the capability to perform operando measurements on full devices on two complementary synchrotron beamlines. The data acquired reinforces the need to elucidate performance–chemical–structural interactions to fully understand degradation processes in order to ultimately stabilize these promising lighting technologies.

Throughout the beamline developments and device studies, we used PeLEDs with deep blue emission around 464 nm, fabricated according to the recipe empirically optimized from Yuan et al.^9^ with an architecture as follows: glass/indium tin oxide (ITO)/​poly­(9-vinylcarbazole) (PVK)/​poly­(vinylpyridine) (PVP)/​perovskite/2,2′,2″-(1,3,5-benzinetriyl)-tris­(1-phenyl-1*H*-benzimidazole) (TPBi)/​8‑hydroxy­quinolinato lithium (Liq)/Al (Figure 1a, with device cross-section SEM image shown in Figure 1b). The perovskite emitter is mixed-halide CsPb­(Br_0.6_Cl_0.4_)_3_ with the addition of the organic 4-fluoro­phenyl­ethyl­ammonium bromide (*p*-FPEABr), to induce the formation of 2D/3D phases that aid the emission through charge carrier funneling, and the addition of other halide salts FABr, LiBr, and ZnCl.^33^

**Figure 1:**
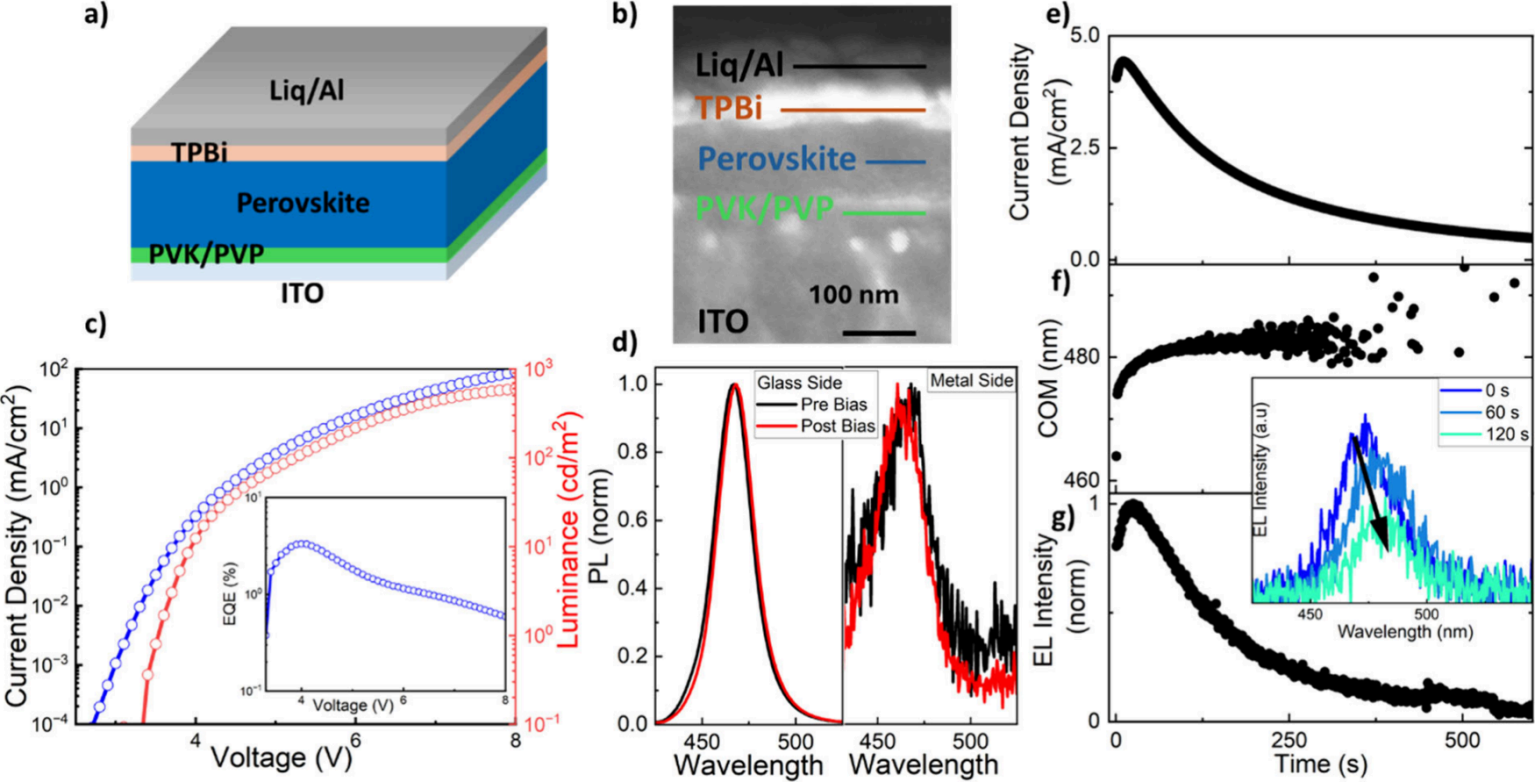
a) Device architecture of the blue PeLED. b) Cross-sectional SEM micrograph of the blue PeLED. c) Current density–voltage–luminance characteristics of the champion deep blue PeLED. Inset: corresponding EQE vs voltage characteristic. d) Normalized PL spectra of PeLEDs before (black) and after bias (red) taken from the glass side and through the metal electrode, with excitation at 405 nm and excitation intensities of 267 mW/cm^2^ and 24 W/cm^2^ for the glass and metal sides, respectively. To achieve the PL signal, the aluminum thickness on the active area was reduced to 50 nm. The PeLEDs were biased for 10 min at 4.5 V. e–g) Optoelectronic data of the PeLED: current density, spectral center of mass (COM), and EL intensity recorded over 10 min at constant applied 4.5 V bias. Inset: selected EL spectra at denoted time snapshots.

Figure 1c shows the current and luminance over voltage performance representative of the deep blue LEDs under investigation, and the inset reflects the corresponding EQE curve. Our champion device reached a peak EQE of 3.3% at a luminance of 12 cd/m^2^ and a turn-on voltage (1 cd/m^2^) at 3.5 V, comparable to reported values for deep blue (<465 nm) PeLEDs without passivation.^8,9,34^ Figure 1e depicts current density data, and Figure 1g shows the normalized EL intensity of a PeLED under constant applied bias (4.5 V) for 10 min. In both graphs we observe a brief initial overshoot, followed by a decay. This same behavior can be seen over numerous PeLEDs (Figure S1). The initial overshoot, commonly observed in many devices, indicates ion migration to the perovskite/transport layer interfaces. This charge accumulation can cause band bending and reduce the injection barrier, which we propose is why we also register a current density increase, from 4 to 4.4 mA/cm^2^, in the same time window.^22,35^ The normalized EL intensity then drops to its half-life time (T_50_) in roughly 104 s of operation. The EL intensity loss is irreversible after 10 min of continuous bias (Figure S2), indicating that at these times scales and operation conditions there is too much damage for the device to recover.

This EL intensity decay is accompanied by color instability as the initial EL emission wavelength with center of mass (COM) around 464 nm rapidly red shifts beyond 480 nm (Figure 1f). To understand this observation further, PL measurements of as-made (pristine) and operated PeLEDs under the same conditions were taken both from the glass side and through the aluminum contact (Figure 1d and Figure S3). The spectra from the glass side display a small red shift of the COM, from 465 to 468 nm. By contrast, data collected from the opposite side reveal a COM blue shift from 463 to 461 nm after applied bias. This result is consistent with a relatively higher fraction of chloride than bromide near the top (metal side) of the device, causing a blue-shifted PL emission. This agrees with another study, where chloride ions were revealed to accumulate in pre-existing defective regions within the perovskite film, leading to a blue shift of the PL in degraded PeLEDs despite a red-shifted EL spectrum.^23^ This result motivates further investigation and puts the focus on the role of interfaces within the device architecture.

To investigate possible structural and interfacial changes during the degradation of the PeLEDs, we measured operando GIWAXS on devices with the schematic shown in Figure 2a. A photograph of the purpose-built sample stage is displayed in Figure S4. The sample holder used in our previous work focusing on photovoltaics^30^ was modified to include an optical fiber connected to a spectrometer that permitted us to record emission spectra of the PeLEDs under operational bias, thus allowing simultaneous collection of electrical, optical, and structural data. Figure S5 shows the modified PeLED layout necessary for operando GIWAXS measurements. This being a grazing X-ray technique and therefore surface focused, we optimized the device architecture to ensure that the perovskite layer was correctly probed. First, we changed the aluminum thickness in the device stack, reducing it from 100 to 50 nm in the active emitting area to improve the X-ray transparency of the metal contact; we see slightly faster performance degradation in such a configuration but the qualitative trends are similar (Figure S16). While it is still possible to probe beneath a thicker metal electrode layer when going above its critical angle,^36^ we risk losing potentially important information on those layers closer to the top interface which are likely to be of crucial relevance.^37^ To ensure proper electrical connection, we deposited 100 nm of aluminum outside of the active area where the electrical contact is made. Second, due to the long X-ray beam footprint at grazing angles,^38^ we fabricated our PeLEDs with a large enough area to ensure that we were only and always probing the actively emitting section of the substrates. A summary of the parameters of the PeLEDs for GIWAXS measurements is reported in Table S1.

**Figure 2:**
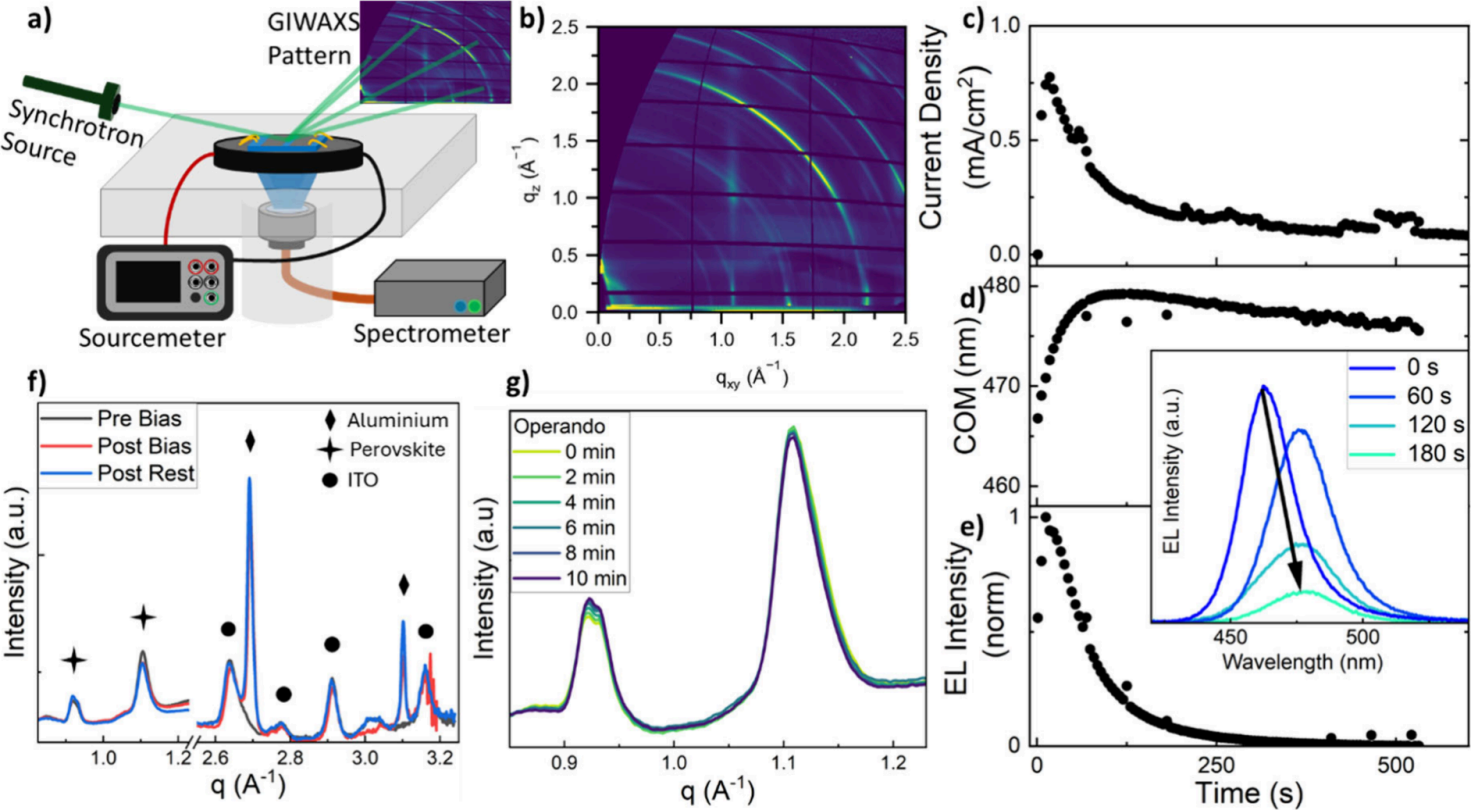
a) Schematic of the operando GIWAXS setup. b) Example of a GIWAXS scan taken at 0.18° pitch angle of the control deep blue PeLED. c–e) Optoelectronic data of the PeLED: current density, COM, and EL intensity recorded over 10 min at constant applied 4.5 V bias. Inset: selected EL spectra at denoted time snapshots. f) 1D Line integration GIWAXS scans taken at 0.4° pitch angle prebias (black), postbias (red), and after rest (blue). g) 1D line integrations of the operando GIWAXS scans highlighting the perovskite peaks going from 0 min (light green) to 10 min (dark blue). Scans were taken at a fixed 0.18° angle.

We acquired GIWAXS patterns through the metal contact, scanning the pitch from 0.04° to 0.4° before and after applying constant bias and then after 10 min of rest. Patterns at selected pitch angles of the depth profile taken before operando measurements are shown in Figure S6, along with the corresponding integrated 1D profile (Figure S7). Figure 2b depicts a GIWAXS scan at a 0.18° pitch angle taken on the deep blue device. Due to the presence of the organic cation spacer p-FPEABr in the precursor solution, there is evidence of a certain degree of structural orientation, represented by the peak at 0.35 Å^–1^ and the vertical streaks appearing at *q*-values around 1.1, 1.5, and 2.15 Å^–1^. Such a scattering pattern indicates a quasi-2D system with in-plane orientation. The 1D line integrations of GIWAXS patterns taken at 0.4° are shown in Figure 2f. We highlight the coexistence of two different perovskite crystal structures in our devices: the larger peak at 1.104 Å^–1^ is assigned to the CsPb­(Br_0.6_Cl_0.4_)_3_ orthorhombic phase, while the two close peaks at 0.918 and 0.931 Å^–1^ belong to the Cs_4_Pb­(Br_0.6_Cl_0.4_)_6_ trigonal phase, consistent with the structure reported by Yuan et al.^9^

The first depth-profile scan was taken just outside of the edge of the PeLED, while the operando measurements and the following two depth-profile scans were all then performed on the PeLED, as confirmed by the appearance of the aluminum peaks at 2.69 and 3.1 Å^–1^. Throughout the measurements, we utilized the 2.15 Å^–1^ ITO peak as a reference to track any temperature changes or drift in the system. Figure S8 shows the full 1D profile and a zoomed-in view of the ITO reference peak. Additionally, we report the optoelectronic data of the PeLEDs recorded simultaneously. Figure 2c shows the device current density that decreases over time. The same trend is also seen in Figure 2e for the EL intensity, which reaches T_50_ in 63 s, and the coupled COM peak emission shift is also shown in Figure 2d as it moves from 466 to 475 nm. Overall, the observed trends are qualitatively similar to those reported in Figure 1, including an overshoot in both current density and EL intensity, giving confidence that the adapted device architectures remain representative of optimized devices.

For the operando measurements, the mounted devices were kept at 4.5 V applied bias while GIWAXS scans were simultaneously taken every 1 min at a 0.18° fixed pitch angle for 10 min in total. We note that the pitch angle was fixed, as changing the angle during the measurement caused misalignment, and 0.18° was specifically chosen for the best signal-to-noise ratio for the perovskite signal. Figure S9 reports both the full line integrations of the operando scans and the highlighted ITO peak; this last one does not exhibit any peak shift, indicating that there is no significant temperature variation, sample drift, or misalignment during the measurements (see Figure S10 for the corresponding GIWAXS images). The operando diffraction data shown in Figure 2g reveals that the overall perovskite crystal structure does not undergo significant changes while bias is applied, and the peaks we observe are similar to those obtained from the scans before operation. From the scan series in Figure 2g, we register a small intensity signal increase of the trigonal peaks at 0.918 and 0.931 Å^–1^, accompanied by a small intensity signal decrease in the orthorhombic peak at 1.104 Å^–1^. We also observe a decrease of the fwhm (from 0.05 to 0.04 Å^–1^) of the same 1.104 Å^–1^ peak with no shift, suggesting a possible phase transition in which portions of the polycrystalline film evolve from the latter to the former. This trend is also detected at smaller pitch angles when the scans are compared before and after electrical operation (Figure S11). The intensity signal variation is also recurrent in peaks at higher *q*-values of the trigonal phase at 1.609, 1,967, 2.045, and 2.067 Å^–1^ and of the orthorhombic phase at 1.542, 1.827, 1.847, 1.927, and 1.983 Å^–1^ (Figure S12). The peaks and structural motifs assigned to the 2D spacer also do not show variations based on the diffraction measurements. We also observe similarly small changes in the structural properties when employing a slightly different composition (Figure S13). Thus, from the operando GIWAXS measurements, we observe that little change happens to the perovskite structure, even though more evident failure happens to the optoelectronic performance of the device.

We then investigated the observed PeLED degradation with an even more surface-sensitive technique, namely HAXPES, in which we explicitly probed the top contact interface (probing depth of approximately 30 nm, see Table S2). For these experiments, the LED architecture had to be modified further with even thinner metal electrodes (15 nm) as well as a thinner top transport layer (15 nm TPBi) to facilitate the escape of photoelectrons from the device in the HAXPES experiment. A photograph and diagram of the modified PeLEDs and sample holder are reported in Figures S14 and S15. The devices were fabricated with a larger area compared with in-house PeLEDs to make sure the X-ray beam spot was contained entirely in the active emitting section of the substrate. A summary of the parameters and thicknesses used for HAXPES PeLEDs is reported in Table S1. Samples were loaded on a compatible holder, which was modified to guarantee electrical contact without hindering the measurement. However, in contrast to GIWAXS, here it was not possible to track the EL spectra emitted during applied bias. Figure 3a illustrates a schematic of the operando HAXPES setup utilized in which the ITO/anode electrode was grounded.

**Figure 3:**
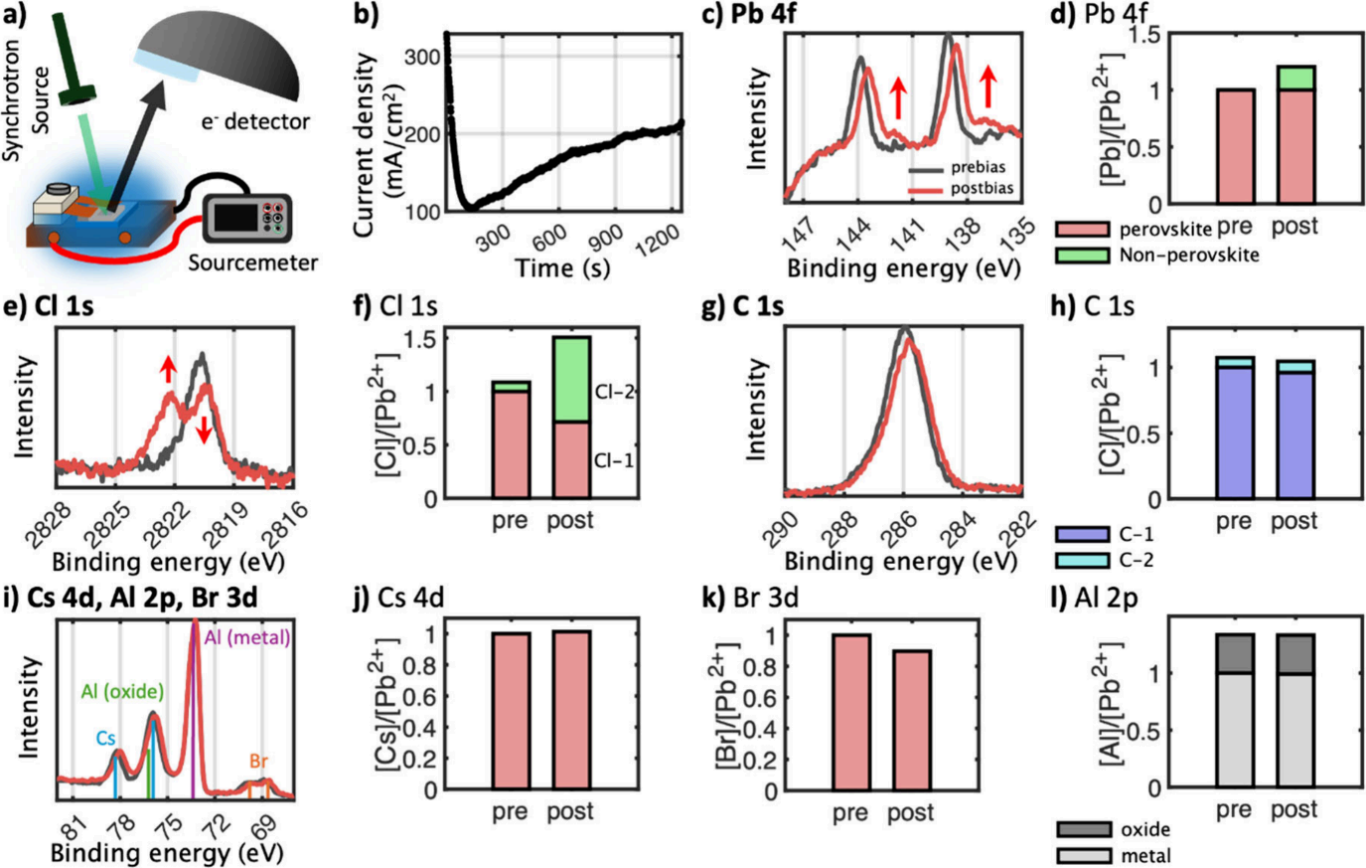
a) Diagram of the operando PES setup. In this configuration, the ITO/anode electrode was grounded. b) Current density over time recorded during 20 min of constant applied bias at 4.5 V. (c,e,g,i) Core-level spectra (Pb 4f, Cl 1s, C 1s, Cs 4d, Al 2p, Br 3d) recorded at 6.6 keV before (black) and after (red) biasing at 4.5 V for 20 min. All spectra were energy calibrated against the Fermi level recorded on an Au foil mounted on the manipulator and intensity normalized to Pb 4f (Pb^2+^). d,f,h,j–l) Next to each core-level spectrum are shown the corresponding compositional changes relative to Pb 4f (Pb^2+^) with perovskite components (red), nonperovskite components (green), primary (purple) and secondary (blue) carbon species, aluminum metal (light gray), and aluminum oxide (dark gray).

To gain information on compositional changes caused by biasing while excluding X-ray-induced changes, HAXPES spectra were first recorded before and after biasing devices at 4.5 V for 20 min. Figure 3a shows the current density over time during the biasing. We observed an initial overshoot up to 329 mA/cm^2^, followed by a quick decay down to 103 mA/cm^2^, after which we registered a gradual increase again toward 210 mA/cm^2^. The J-V data also reflects a similar initial overshoot trend followed by breakdown, as seen in Figure 1e and Figure 2c. The ultrahigh vacuum conditions of the measurement likely accelerate degradation mechanisms^39^ and are consistent with the observed faster drop in parameters compared to in-house/GIWAXS (Figure S16). This may explain the very sharp current breakdown we observed here, after which the LED loses its rectifying nature and becomes less resistive. Nevertheless, the data shows that a current flow was detectable throughout the entire duration we biased the LED. The functionality of the PeLEDs was further confirmed via a microscope camera coupled to the analysis chamber, confirming that the PeLEDs initially emit light (Figure S17). However, the light quickly dimmed after a few minutes, i.e., on the same time scale as the initial drop in current. We note that we attribute the different magnitudes between the current densities throughout the various PeLED layouts to the different pixel areas and transport layer thicknesses, which are summarized in Table S1 and explained further in the SI. Specifically, for HAXPES, a lower thickness of the electron transport layer (ETL) induces more current leakage, which would contribute to the higher values registered for those device structures as well as instigating a quicker device breakdown (Figures S15 and S16).

In order to quantify X-ray-induced changes to the perovskite, we recorded three consecutive measurements of the core-level spectra (Figures S18 and S19). Shifts in binding energy of ∼0.3 eV were observed for the Pb 4f and Cl 1s core levels after the first measurement, both before and immediately after biasing. As these were observed only after the first measurement, the shifts were attributed to some minor X-ray-induced electronic changes of the perovskite surface upon initial X-ray illumination. However, the core-level spectra subsequently stabilized, and after two consecutive scans no changes in relative core-level intensities or shape were observed, indicating no significant X-ray degradation is present after the initial scan. Therefore, we attribute changes in the spectra before and after biasing to the effect of biasing rather than X-ray illumination.

Figure 3c,e,g,i shows the averaged photoelectron core-level spectra before (prebias, black) and after biasing (postbias, red). The spectra were intensity-normalized to the Pb^2+^ contribution of the Pb 4f core-level peaks to obtain information relative to the perovskite structure. Curve fitting was carried out on all spectra to quantify the compositional changes before and after biasing relative to Pb^2+^ (curve fits are reported in Figures S20 and S21). The quantification results are shown in Figure 3d,f,h,j–l next to their respective spectra. The contributions from the perovskite constituents, the main carbon contribution, and the aluminum metal contribution before biasing were scaled to 1, and the composition changes after biasing are shown relative to the prebiased composition.

The perovskite components show substantial changes in their core-level spectra after biasing compared with before biasing. Core-level peaks were assigned using reference spectra of the perovskite, of the perovskite with the ETL on top, of the ETL layers, and of the aluminum (Figures S22–S25). First, 20% metallic lead (Pb^0^at 137.0 eV) formed after biasing, indicating degradation of the perovskite (Figure 3c,d). Furthermore, two chlorine species were visible from the start (Figure 3e,f), a perovskite-related species at lower binding energy (2820.4 eV, “Cl-1”), and a small amount of a nonperovskite-related species at higher binding energy (2822.2 eV, “Cl-2”) (assignment based on comparison to Figure S22). After biasing, the perovskite chloride species at lower binding energy decreased while the nonperovskite species at higher binding energy increased substantially. This could be related to the nonperovskite species approaching the surface, the perovskite species moving toward the bulk, or a combination of both, and to a transformation of the perovskite species into a product related to the nonperovskite chlorine species. The shift to higher binding energy between the perovskite Cl and the nonperovskite Cl is in agreement with nonperovskite Cl being in a higher oxidation state than the Cl within the perovskite, where it has an oxidation state of −1. Alternatively, the higher binding energy can suggest more electron-withdrawing surroundings of the chlorine species, which would suggest that this new chlorine species is not within the perovskite anymore, as we will also notice from the operando measurements further below. As there are several factors influencing the binding energy shift, it is difficult to assign with certainty which effects dominate here. However, it is clear that the nonperovskite chlorine species has a lower electron density than the perovskite chloride.

A slight increase in the Cs 4d features (Cs 4d_5/2_ at 75.85 and Cs 4d_3/2_ at 78.15 eV) and a small decrease in Br 3d peaks (Br 3d_5/2_ at 68.8 and Br 3d_3/2_ at 69.85 eV) are also observed (Figure 3i,j,k); i.e., cesium may move toward the surface while bromide may move toward the bulk, which could be consistent with the PL red shift on the other side of the film reported in Figure 1d. Additionally, both bromide and the perovskite chloride species may form gaseous degradation products and leave the device structure entirely, as has previously been reported.^19^ In this case, the formation of metallic lead together with an increased presence of cesium and nonperovskite chloride species suggests that the perovskite composition has changed. Overall, the observed changes indicate some bias-induced degradation of the perovskite material. The C 1s spectra, corresponding to the TPBi electron transport layer, show slight changes in intensity (Figure 3g,h). Two contributions were observed for the C 1s spectra, one at lower binding energy (285.8 eV, “C-1”) and one at higher binding energy (287.3 eV, “C-2”). The C-1 contribution decreased, while the C-2 contribution increased slightly. We note that there may be additional contamination at the aluminum surface. Additionally, the organic cation used as a spacer material in the perovskite also contains carbon, further complicating the fitting and interpretation of the carbon signal. The aluminum metal peak (73.2 eV) and the aluminum oxide peak (76.0 eV) intensities did not change significantly upon biasing (Figure 3i,l). Notably, the Pb 4f spectra and the C 1s spectra are shifted toward lower binding energies after biasing.

To provide further insight into the degradation components found after biasing, HAXPES spectra were recorded on the same device while simultaneously biasing (i.e., measuring operando) and again after biasing. This allows us to follow the electrical field buildup throughout the device and understand where the different species are spatially, i.e., closer toward the surface or farther in the bulk. Figure 4a–d shows representative core-level spectra of the different components (Pb 4f, Cl 1s, C 1s, Al 2p, Br 3d, and Cs 4d) of the PeLED during biasing (blue) and after biasing (red), with corresponding raw core-level spectra reported in Figure S26. While all spectra show a shift toward lower binding energies under biasing due to the potential at the different layers, not every component shifts by the same amount. We note that the main C 1s peak splits into two contributions under biasing. This suggests that the carbon signal is observed from different layers within the structure, which experience a different fraction of the applied bias. Figure 4e shows the shifts of all core levels, sorted by shift magnitude. We note that the Cl 1s, C 1s, and Al 2p (oxide) peak positions have a higher error range, as they show overlapping peaks either during (Cl 1s) or after biasing (C 1s, Al 2p (oxide)). As expected, the core levels relating to the top electrode where bias is applied, i.e., the aluminum metal and oxide core levels, shift the most. This is followed by part of the main C 1s contribution (“C-1A”), the non-perovskite Cl 1s species at higher binding energy (“Cl-2”), the perovskite Cl 1s component at lower binding energy (“Cl-1”), and the secondary C 1s component (“C-1B”). The other perovskite contributions shift the least and by similar amounts (Pb 4f, Br 3d, Cs 4d). Repeated samples showed similar trends (Figure S27). The Pb^0^ shift could not be fitted in these measurements due to its small intensity and the small difference in binding energy shift under bias compared to the Pb^2+^ component. Similarly, the C-2 contribution was not fitted due to its small intensity. We also note that the aluminum metal shifts by less than the applied bias of 4.5 V, indicating some electrical losses from the experimental setup. We note that the degradation caused by previous biasing could also affect the maximum shift observed.

**Figure 4:**
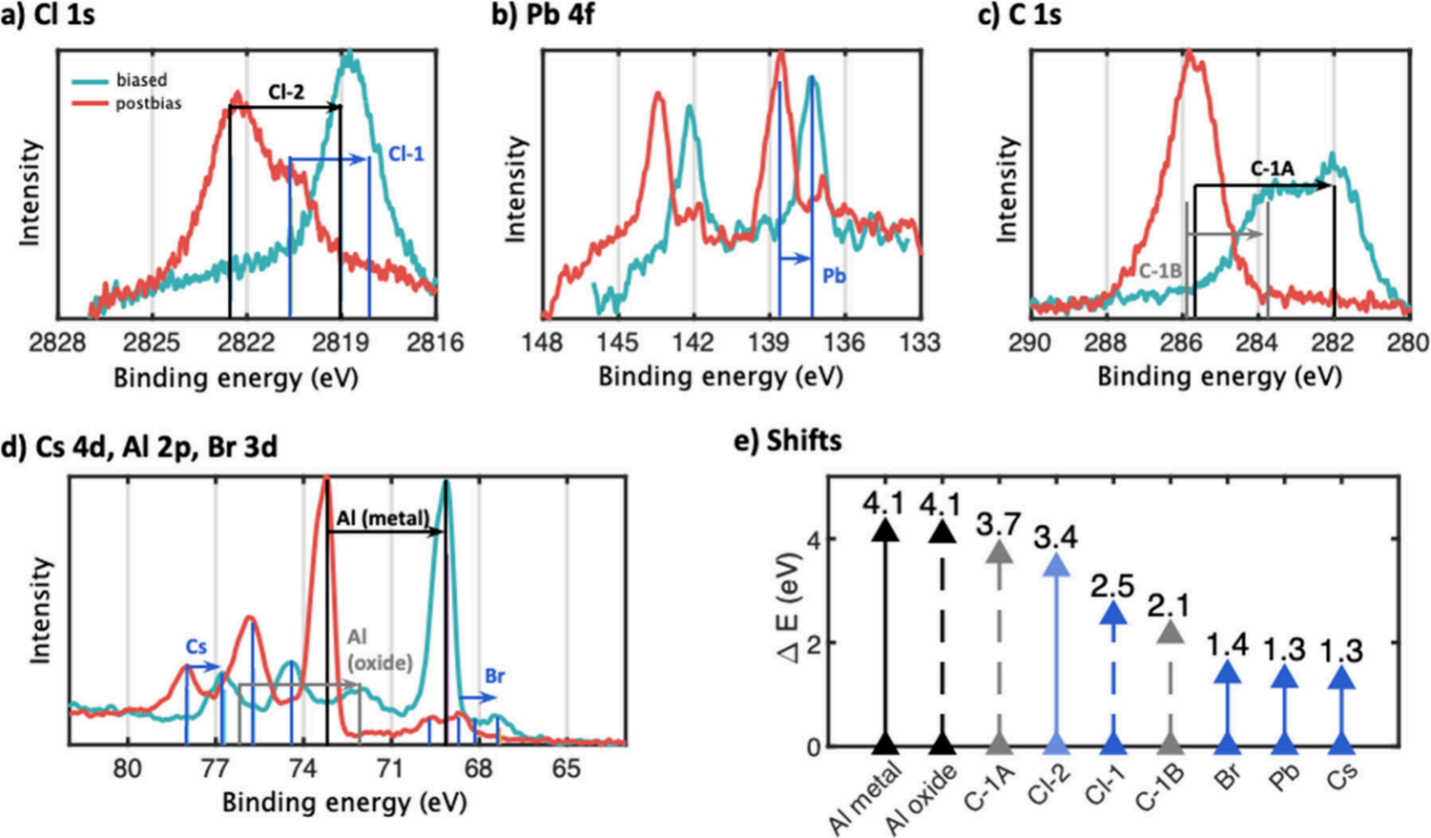
a–d) Core-level spectra (Cl 1s, Pb 4f, C 1s, Cs 4d, Al 2p, Br 3d) recorded at 6.6 keV while biasing at 4.5 V (blue) and after biasing (red). The aluminum background was subtracted from the Cl 1s and Pb 4f spectra. All spectra were energy calibrated against the Fermi level recorded on an Au foil mounted on the manipulator and intensity normalized toward the Pb^2+^. e) Shifts in binding energy for each core level sorted by their magnitude.

The different magnitude of the shifts suggests that the nonperovskite Cl 1s species at higher binding energy (Cl-2) is closer to the aluminum contact than the other Cl 1s species at lower binding energy (Cl-1) or any other perovskite species, as its signal shifts significantly more under the bias than the rest. Specifically, the Cl-2 shift of 3.4 eV is more similar to the shift of C-1A (3.7 eV) than to the shifts of the perovskite core levels Pb 4f, Cs 4d, and Br 3d (1.3 to 1.4 eV), while Cl-1 shifts by 2.5 eV. This suggests that the Cl 1s species at lower binding energy remains within the perovskite structure, while the Cl 1s species at higher binding energy is not in the perovskite but rather within the TPBi layer. Part of the degradation therefore involves the movement of chloride ions out of the perovskite layer and toward the aluminum contact. Such a movement agrees with the overall increase in chloride signal intensity, as species in the top layers will be closer to the sample surface than the species in the perovskite layer and therefore show a relatively higher intensity in the photoelectron spectra. Overall, these results reveal the presence of an electric field gradient across the different device layers. Notably, we see a big voltage drop at the top interface, meaning that the perovskite components underneath are affected differently. While the Pb 4f, Br 3d, and Cs 4d core levels shift by roughly the same amount, the perovskite Cl 1s core level at lower binding energy (Cl-1) shifts more. As the two Cl 1s contributions overlap under bias, the peak position shift of Cl 1s has a higher uncertainty than the peak shifts of the other perovskite components. Furthermore, measurements of the Cl 1s core level have a substantially higher surface sensitivity in PES than the other perovskite core levels (Table S2). Both factors could contribute to larger shifts compared with the other perovskite core levels.

Lead halide perovskites are known to degrade when in contact with metals,^40,41^ which may be more readily triggered by our device design for the HAXPES operando measurements, in which the organic TPBi top contact is very thin compared to the other device configurations. Therefore, further measurements on PeLED reference samples with varying TPBi thicknesses were carried out (Figure S28). For these samples, Pb^0^ was observed for the thinner TPBi layers without applying an external bias. A clear trend is visible, where thinner TPBi correlates with larger amounts of Pb^0^ formation and greater Cl 2s total intensities. We note that these samples were measured during a successive beamtime, and the TPBi thicknesses may be slightly thinner compared to the one used in the PeLEDs reported in Figures 3 and 4. The PeLED with the thickest TPBi layer (nominal 30 nm) shows no Pb^0^ and generally a much smaller Cl 2s intensity. This suggests that proximity with metals facilitates degradation, similar to observations in perovskite solar cells.^40,41^ Therefore, strategies to limit the contact, such as sufficient TPBi layer thickness in these devices, may improve device stability while also preventing the formation of preexisting degradation sites which can act as degradation seeds.^23^ The thinner layers used in the PES measurements may accelerate degradation compared to typical device architectures due to enhanced electron injection and therefore more Pb^2+^ reduction. However, the results collected are still relevant, as PeLEDs with larger TPBi and aluminum thicknesses, as used in lab tests (Figure 1e–g and Figure S16), follow similar degradation trends, albeit at slower rates and with lower current density values.^42,43^

We can now draw qualitative conclusions on device degradation and a generalized degradation mechanism for these deep blue PeLEDs, as depicted in Figure 5.

**Figure 5:**
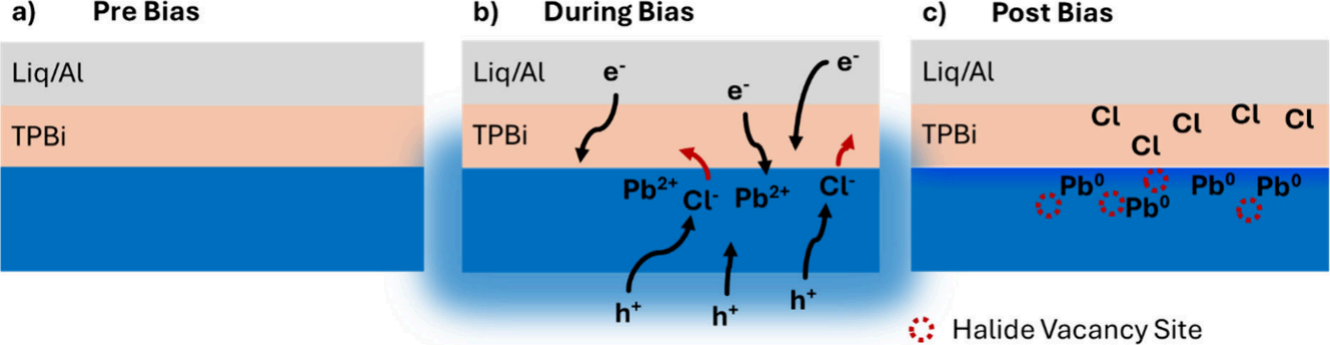
Proposed degradation mechanism occurring in deep blue mixed Br/Cl PeLEDs. a) Blue PeLEDs before any applied bias. b) During operation, electrons and holes are injected into the perovskite and a field is established, leading to halide segregation and migration, driven in particular by halide oxidation. This process also exposes more uncoordinated Pb^2+^ ions, which can be reduced by the electrons. c) Postbias, at the top interface with the ETL, we find metallic lead concomitant with the new Cl species (Cl-2) that sits outside of the perovskite structure, attributed to atomic chlorine, along with other halide vacancies.

Our electrical data show clear device degradation, and the GIWAXS results suggest that the perovskite bulk structure remains largely intact, while the HAXPES results reveal substantial changes of the perovskite top interface, including Pb^0^ formation and a new chlorine species, which is present in the contact layers above the perovskite. As this chlorine species is observed at a higher binding energy than the perovskite chloride, it has a lower electron density than the perovskite chloride; this may be, for example, oxidized chloride (atomic chlorine) (Figure 5c).^44^

We note that, with the HAXPES measurements here and the extra layers on top, we can only collect information on the surface and are not able to track changes farther into the bulk and toward the HTL side. Furthermore, due to the sensitivity of HAXPES, even a small amount of Cl migration from the perovskite to the TPBi will result in a strong signal of the halide within the ETL (Figure S29). At the same time, GIWAXS is not able to detect small interfacial changes in the perovskite due to the presence of the ETL and metal, which means that at small angles the TPBI and Al signals mix with scattered X-rays from the surface of the perovskite. During biasing (Figure 5b), the charge injection into the perovskite and/or fields applied across the device causes chloride ions to oxidize, reach the top interface, and even penetrate into the ETL and toward the electrode while bromide migrates away from the top interface. Halide migration across the perovskite emitter toward the top interface is also consistent with the PL results from Figure 1d. This leaves undercoordinated Pb^2+^ behind, which then reduces to Pb^0^ due to the presence of injected electrons, particularly at that top interface. After bias (Figure 5c), the region close to the top interface now contains metallic Pb and halide vacancies due to ion migration. The critical role of chloride is further validated by the comparison of our deep blue mixed Cl/Br PeLEDs with pure Br systems, where we see a slower decay, particularly in EL intensity (Figure S30). Additionally, our results on devices with varying TPBi thickness (Figure S28) show that thinner TPBi layers cannot protect the perovskite sufficiently, which leads to similar degradation reactions happening without applying bias and could contribute to accelerated degradation under bias as well.

A recent study on sky blue PeLEDs with architecture similar to ours found evidence of halide segregation and migration.^45^ In particular, the researchers found an increase in chloride toward the Al/LiF/TPBi top interface after biasing the devices. Additionally, they tested inverted PeLEDs with ZnO as the ETL and found also here chloride migration to the ETL dominating after biasing; i.e., the chloride migration was in both cases to the cathode side. This agrees with our results, where we found a decrease in perovskite chloride species together with an increase in a nonperovskite chlorine species which is spatially closer to the metal contact than the perovskite chloride species. In addition to this, our results showed formation of Pb^0^, indicating further degradation of the perovskite material that likely results from the halide movement. Our results show that the severity of degradation is dependent on the TPBi thickness. Therefore, working on preventing these interfacial chemical reactions is necessary to improve device stability. Furthermore, there should still remain a focus on also stabilizing the bulk perovskite, including its phase stability, against any long-term structural changes that may occur. Learning from the photovoltaic community, possible strategies to explore and apply to PeLEDs include integration of interfacial 2D layers, thin buffer layers, or perovskite surface functionalization to protect and passivate the emitter at the charge transport interfaces.^46–48^ Another route is to explore alternative device architectures that minimize the problematic halide segregation and ion migration. Knowing that chloride ions are prone to move around the perovskite structure more easily than the other halide species, strategies to fix them in place within the perovskite octahedra would benefit blue PeLEDs immensely.^22^

We investigated deep blue PeLED degradation and operational stability through the development of two operando synchrotron-based techniques, GIWAXS and HAXPES. Being able to perform these measurements on full stack devices while they are operating and emitting light adds a new dimension to the recorded data, as it is possible to link trends between diffraction and photoemission results with the optoelectronic performances and chemical changes. Photoemission spectroscopy reveals ion migration from the perovskite emitter into adjacent layers when bias is applied, and this is reflected in the initial increase of both EL and current density. The scattering patterns reveal the coexistence of two perovskite phases and a small transition from orthorhombic to trigonal during applied bias but overall only very small changes in structure. Simultaneously, current density, EL intensity, and spectral degradation occur in the PeLED, showing the same current and EL intensity overshoot followed by a decay trend. It is only with HAXPES measurements that we can track the ion migration at the interface occurring inside the blue emitter while it degrades, as we report the formation of a new chloride species that leaves the perovskite structure and migrates toward the aluminum metal contact as well as bromide ions moving away from the top interface. As a consequence of the resulting halide vacancies, the under-coordinated lead is reduced and forms metallic lead. The evolution of these already established techniques opens a new avenue for the study of full stack PeLEDs’ deterioration and instability. This study further highlights the importance of preventing reactions between the perovskite and the metal and preventing halides from migrating to the interfaces, as our results show a clear dependence on ETL thickness on the observed degradation. Future studies must focus on addressing this problematic interface to avoid premature degradation and advance the development of bright, efficient, and stable deep blue PeLEDs. The techniques developed here are now available for users on Diamond Light Source I07 (GIWAXS) and I09 (HAXPES) beamlines, and we expect future work will allow insights into new compositions of deep blue PeLEDs, other types of PeLEDs including those emitting in the green or red, as well as other emerging lighting technologies.

## Supplementary Material



## Notes

Materials, solution and device preparation, film and device characterization, GIWAXS and HAXPES methodology, J-V curves and electrical stability measurements, PL data, GIWAXS sample stage photographs and device schematics, complementary GIWAXS data, HAXPES sample stage photographs and device schematics, complementary HAXPES data, and ETL thickness analysis (PDF)
