# Supplementary material for: Interfacial Chemistry Limits the Stability of Deep Blue Perovskite LEDs Revealed by Operando Characterization
Source: ACS Energy Lett. 2025 Jun 28;10(7):3533–43. doi: 10.1021/acsenergylett.5c00989 (PMC12261314; doi:10.1021/acsenergylett.5c00989)
Supplement: Supplementary file 1 [file nz5c00989_si_001.pdf]

Interfacial Chemistry Limits the Stability of Deep Blue Perovskite LEDs Revealed by Operando Characterization

Alessandro J. Mirabelli[1,2,#], Birgit Kammlander[3,#], Yang Lu[1,2], Rahul Mahavir Varma[3], Qichun Gu[1], Karen Radetzky[3,4], Thomas A. Selby[1], Tianjun Liu[2], Stefania Riva[3], Zimu Wei[1], Tien-Lin Lee[5], Jonathan Rawle[5], Håkan Rensmo[3,4], Miguel Anaya[1,6], Ute B. Cappel[3,4] Samuel D. Stranks[1,2*]*

[1]Department of Chemical Engineering and Biotechnology, University of Cambridge, Philippa Fawcett Drive, Cambridge CB3 0AS, UK

[2]Cavendish Laboratory, University of Cambridge, JJ Thomson Avenue, Cambridge CB3 0HE, UK

[3]Division of X-ray Photon Science, Department of Physics and Astronomy, Uppsala University, Box 516, 751 20 Uppsala, Sweden

[4]Wallenberg Initiative Materials Science for Sustainability, Department of Physics and Astronomy, Uppsala University, 751 20 Uppsala, Sweden

[5]Diamond Light Source, Harwell Science and Innovation Campus, Didcot, OX11 0DE, UK

[6]Departamento de Fisica de la Materia Condensada, Instituto de Ciencia de Materiales de Sevilla, Universidad de Sevilla-CSIC, Avenida Reina Mercedes SN, Sevilla 41012, Spain

[#]The authors contributed equally to the work.

*email: ute.cappel@physics.uu.se, sds65@cam.ac.uk

Experimental Section

Materials

Caesium bromide (CsBr), lead bromide ($PbBr_2$), lead chloride ($PbCl_2$), lithium bromide (LiBr), zinc chloride (ZnCl), PVP, PVK (molecular weight 25,000 – 50,000 g mol^{-1}), dimethylsulfoxide (DMSO) (anhydrous 99,9%), isopropanol (IPA) and chlorobenzene (CB) were purchased from Sigma-Aldrich. Formamidinium (FABr) was purchased from Greatcell Solar, p-FPEABr was purchased from Xi'an Polymer Light Technology, TPBi and Liq were purchased from Ossila, Aluminium was purchased from Kurt J. Lesker. All materials were directly used without further purification.

Precursor Solution Preparation

The perovskite precursor solution for deep blue EL emission was prepared by dissolving p-FPEABr, CsBr, FABr, LiBr, ZnCl, and ($PbCl_2$ + $PbBr_2$) in DMSO with molar ratio of 0.80:1.00:0.05:0.30:0.05:0.95. A CsBr concentration of 0.12 M was maintained.

To achieve deep blue emission, the molar ratio of $PbCl_2/PbBr_2$ was 2:1. The solution was stirred for 2 h before use. PVK was dissolved in CB at a concentration of 6 mg/ml. The PVP precursor was dissolved in IPA at 2 mg/ml. Both perovskite solutions were filtered using a 0.45 µm polytetrafluoroethylene (PTFE) filter before use.

Device Fabrication

ITO-patterned glass substrates were sequentially cleaned with water and soap, water, acetone and IPA for 15 minutes each. Clean ITO substrates were then treated with UV Ozone for 15 minutes before being transferred into a Nitrogen filled glovebox. Devices were fabricated with the following structure of ITO/PVK/PVP/perovskite/TPBi/Liq/Al. The PVK solution was filtered with a 0.2 µm PTFE filter before use. The solution was spin coated onto ITO substrates at 5000 rpm for 35 s, followed by annealing at 100°C for 10 min. Then, a thin PVP layer was deposited by spin coating at 5000 rpms for 35 s, followed by annealing at 80°C. Immediately after, the perovskite solution was spin coated at 4500 rpms for 120 s and then annealed for 5 min at 75°C. After this step, the substrates were transferred to another adjoining nitrogen filled glovebox without exposing to air were the remaining layers (TPBi, LiQ and Al) were deposited by thermal evaporation at a vacuum pressure of $<10^{-5}$ Pa.

Devices measured in house were completed with 35 nm of TPBi, 1.5 nm of Liq and 100 nm of Al. The active area was 0.045 cm^2. Devices for operando GIWAXS measurements had a reduced metal electrode thickness (50 nm) in the active area of the pixel and 100 nm elsewhere. The active area was 0.28 cm^2. Devices for operando HAXPES had a reduced ETL

thicknesses with only 15 nm of TPBi and 1 nm of Liq. The metal electrode thickness on the active area of the pixel was 15 nm and 100 nm elsewhere. The active area was 0.12 cm^2. Variation of the active area impacts the current density produced by the LED based on the formula that returns the resistance of the electrode: $R = \rho\, L/Wt$, where ρ is the resistivity of aluminium, and L, W, t are respectively the length, width and thickness of the pixel. PeLEDs measured in house had pixel dimensions 5.5 x 1.5 mm, the ones used for GIWAXS measurements had pixel dimensions of 12 x 4 mm, while those used for HAXPES had dimensions 5.5 x 4 mm. Following the formula we can see how the current density in GIWAXS PeLEDs would be lower compared to in house devices, due to the much larger increase of the length of the pixel compared to the variation of the width, which overall gives a higher resistance. On the other hand, HAXPES PeLED are only wider and this translates into a lower resistance and higher current density. Thinner transport layers (e.g. TPBi) induce more current leakage through the device leading to higher current densities, while also providing less resistance to charge injection. To achieve different metal thicknesses on the substrates, two different shadow masks where employed and vacuum was broken between each evaporation to change from the first to the second. The various thicknesses are reported below in Table S1.

Table S1: Summary of the thicknesses and areas of the PeLEDs used in the different experiments.

	**Thickness (nm)**		
	**In house**	**GIWAXS**	**HAXPES**
**TPBi**	35	35	15
**Liq**	1.5	1.5	1
**Aluminium**	100	50 (and 100)	15 (and 100)
**Area (cm^2)**	0.045	0.28	0.12

All device fabrication and sample transport as well as most measurements were carried out in inert atmosphere in order to avoid exposure to atmosphere. However, we cannot exclude that in any moment including sample transfer that some oxygen atoms possibly came into contact with our samples and oxidised the aluminium.

Characterization of LED performance

Current density-voltage characteristics were measured using a Keithley 2400 sourcemeter unit. The corresponding photon flux was measured simultaneously using a calibrated silicon photodiode centred over the LED at a distance of 10 cm from the device. The LED metrics were calculated taking into account the responsivity of the photodetector, the geometry of the setup and the electroluminescence spectrum following the guidance of a previous work.[1] The emission spectra were measured with an Ocean Optics spectrometer. The EQE was calculated assuming a Lambertian profile. All devices were encapsulated using Blufixx UV

curing glue and a glass cover. The measurements were carried out in ambient conditions outside of a glovebox.

Operational lifetime measurements were carried out in a N_2 filled glovebox using an integrating sphere connected to a StellarNet Silver Nova spectrometer. The perovskite LEDs were biased using a Keithley 2450 sourcemeter unit. Devices measured here were not encapsulated but transferred from glovebox to glovebox using an hermetically sealed transfer tube in order to avoid exposure to ambient atmosphere.

GIWAXS measurements

The measurements were performed at the I07 beamline of Diamond Light Source facility in Didcot, United Kingdom. The beam energy used was fixed at 10 keV during the measurements and the X-rays were impinging from the metal contact side of the full devices. After alignment, PeLEDs were initially scanned from angles 0.04° to 0.4°, step 0.02°, in the gap between the two pixels (see Figure S5). The exposure time was 1 s throughout the entire set of measurements in order to minimize any possible X-ray damage. After choosing the pitch angle that gave the highest perovskite peak response, the stage was then moved to probe the pixel for operando and successive measurements. The operando data reported in the main text was taken at 0.18°. This angle was chosen because it is above the aluminium critical angle allowing us to penetrate the metal layer and probe underneath. For the operando measurement, GIWAXS patterns were collected once per minute for 10 minutes while the device was held at the desired fixed angle. Immediately after, the applied voltage was turned off and the stage was moved to probe a different spot on the same pixel and another depth profile scan from 0.04° to 0.4°, step 0.02°, was taken. This was followed by 10 minutes of rest in which no x-rays or bias was affecting the LEDs. Finally, one last profile scan again from 0.04° to 0.4°, step 0.02°, pitch angle was taken after moving to a different spot on the pixel. Throughout the entirety of the alignment and measurement, the device chamber was flushed with Helium at 2 L/min.

During the operando scans, the PeLED was biased at 4.5 V for 10 minutes with a Keithley 2450 sourcemeter allowing us to record IV data. Simultaneously, electroluminescence spectra were collected every 5 seconds from the glass side of the devices via a collimator lens coupled to an optical fibre and detected with an OceanOptics Maya2000 Pro fixed-grating spectrometer. The exposure time was 3 seconds.

The devices were mounted on a custom made sample holder, modified from a previous work, [2] that could anchor to the hexapod stage of the beamline therefore permitting us to perform surface sensitive measurements. Compared to the previous work, the stage here was adapted to insert the collimator lens and optical fibre to collect the electroluminescence coming from underneath the PeLED (see Figure S4). The sample holder was made out of steel to dissipate heat and to prevent the PeLED temperature from increasing as much as possible. Post processing of the GIWAXS patterns was performed with an in house written python script. The resulting data was cross checked with the DAWN software. To calibrate the diffractograms in

q values, a LaB_6 GIWAXS pattern was used with sample to detector distance 406 mm. The reference ITO peak at 2.15 $Å^{-1}$ served as peak shift calibration in order to assess correctly any observed differences during the scans. 1D line integrations were performed across the entire quadrant $0 \leq \chi \leq 90$, where χ is the azimuthal angle.

HAXPES measurements

To avoid exposure to ambient air, the samples were transported from Cambridge to Diamond in inert gas filled bags, mounted inside a glovebox at Diamond and transferred to the load lock of the end-station via suitcase. (Operando) PES measurements were carried out at the I09 beamline at the Diamond Light Source (DLS, Oxfordshire, UK) synchrotron facility.[3] The X-rays were monochromatized using a Si (111) double-crystal monochromator. The measurements were carried out at room temperature with sub 10^{-9} bar pressure in the analysis chamber. The photoelectrons were detected via a hemispherical analyser (Scienta EW4000). All measurements were carried out at 6.6 keV photon energy. To avoid beam-induced damage the beam was defocused and the undulator gap was detuned to 8.35 mm.

Surface characterization was done in swept mode at both photon energies and all relevant core levels (Pb 4f, Br 3d, Cs 4d, Al 2p, Br 3p, C 1s, N 1s, Cs 3d, Cl 1s) were measured in loops using a pass energy of 200 eV, a step size of 0.1 eV and a time of integration of 0.176 s. The spectra were energy calibrated to Au $4f_{7/2}$ (84.0 eV) and intensity normalized with respect to Pb 4f.

The operando measurements followed a protocol that allows to distinguish X-ray and bias induced changes. These measurements were carried out at 6.6 keV for selected relevant core levels (Pb 4f, Cl 1s, C 1s, Al 2p, Br 3d, Cs 4d) and measurements were kept as short as possible to avoid any X-ray induced damage using fixed mode. Al 2p, Br 3d and Cs 4d were recorded as one region in fixed mode. Pb 4f and Al 2p were measured with a pass energy of 500 eV and Cl 1s and C 1s were measured with a pass energy of 200 eV. First, the core levels were measured before biasing the device, followed by biasing the device with a Keithley 2634B at 4.5 V without recording PES spectra. Then the PES core levels were recorded post bias without applied voltage. This allowed us to follow any changes induced by biasing only while excluding any X-ray induced changes. Subsequently, the core levels were recorded while simultaneously applying biasing (operando) to follow the core level shifts under operation. The bias was applied on the top metal electrode of the PeLED and bottom ITO electrode was grounded to the same potential as the spectrometer. This allowed us to analyse the electric field in the different top layers of the device. The spectra were normalized to the Pb^{2+} intensity and calibrated to the Fermi level recorded on an Au foil mounted on the sample manipulator. All core level spectra were recorded in loops to follow any changes over the measurement time. An aluminium background subtraction was carried out on the Cl 1s and Pb 4f spectra to improve visibility of the spectra features and ease the analysis.

Curve fitting was carried out in Matlab using pseudo-Voigt functions with a linear, polynomial or Shirley background as seen fit for the surface characterization and core level spectra before and after biasing.[4] For the operando measurements, curve fitting was done with gaussian fits to estimate the peak positions. The greater core level separation of the C 1s and Al 2p (oxide) peaks under bias and of the Cl 1s without bias was used to constrain the gaussian fits in their intensities (see Figure S24).

The inelastic mean free path (IMFP) was estimated using the TPP-2M method, as shown in Table S2.[5] The IMFP was estimated accounting for the aluminium top layer. The parameters used were a density of 2.70 g/cm3, a molecular weight of 26.98 g/mol, 3 valence electrons and no energy band gap. The IMFP ranged from 6.9 to 11.0 nm for 6.6 keV photon energy, depending on the exact core level.

Table S2: Estimation of the inelastic mean free path (IMFP) for the relevant core levels and in dependence of the kinetic energy (E_{kin}) of each core level using the TPP-2M method for a photon energy (E_{photon}) of 6.6 keV. The IMFP was estimated using the top layer aluminium. The probing depth (90% of signal) is given by 3 * IMFP.

**Core level**	**IMFP / nm**	**E_{kin} / eV**
**Cl 1s**	6.9	3779.6
**C 1s**	10.7	6314.3
**Pb 4f**	10.9	6460.9
**Cs 4d**	11.0	6524.1
**Al 2p (metal)**	11.0	6526.8
**Br 3d**	11.0	6531.1

PL measurements

PL spectra were measured using a Teledyne Princeton Instrument HRS-500-SS spectrograph equipped with a Pixis 1024BRX detector. The samples were excited at room temperature with a continuous- wave 405 nm laser source (CNI MGL-III-405-500 mW). The excitation intensity was 24 W/cm^2 when measuring from the metal electrode side and 267 mW/cm^2 when from the glass side.

SEM measurement

SEM was performed on a Zeiss under 3keV accelerating voltage, standard aperture (30 µm) and working distance of 5 mm for optimised morphology characterisation. The cross-section samples were mechanically cut and then sputtered with Au/Pd for 10 seconds to minimize image drifting due to charging under the electron beam.

LED Data

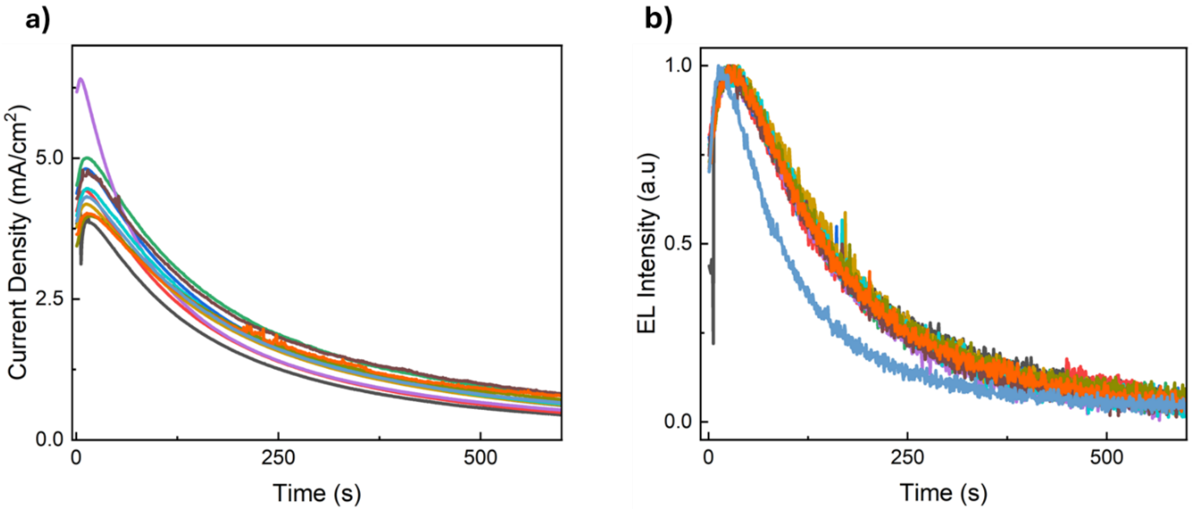

Figure S1: Statistics on 10 devices of current density (a) and normalized EL intensity (b) over 10 minutes at 4.5 V applied bias.

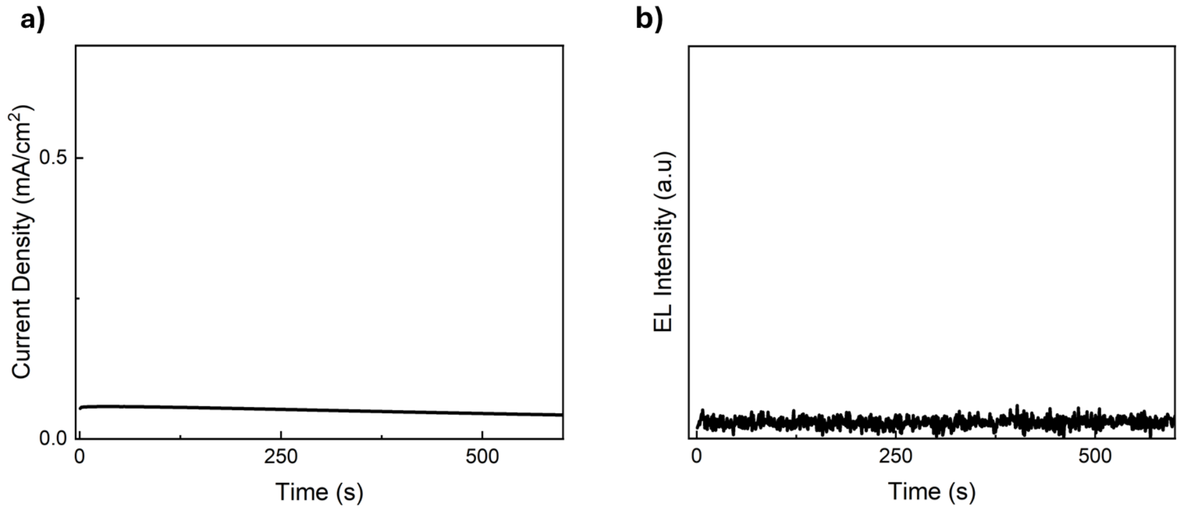

Figure S2: Current over time (a) and EL data (b) of a perovskite LED biased at 4.5 V for 10 min that had already been biased at 4.5 V for 10 min 2 weeks prior, showcasing that the PeLED does not emit any light anymore.

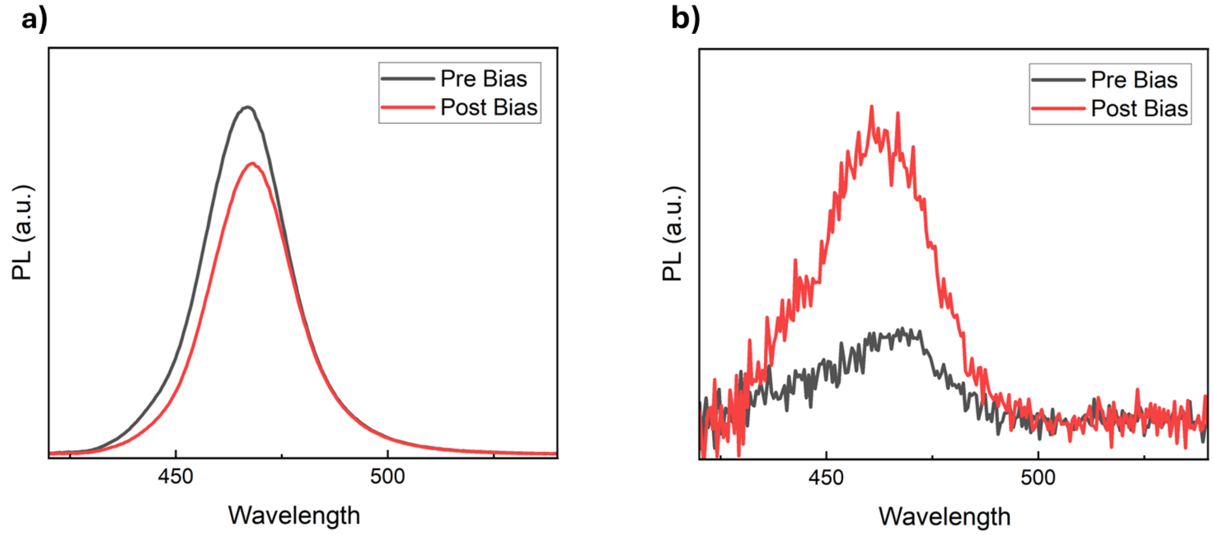

Figure S3: Non-normalized PL data of Figure 1d of PeLEDs taken pre (black) and post (red) bias through the glass (a) and metal (b) side. PeLEDs were biased at 4.5 V for 10 mins.

GIWAXS

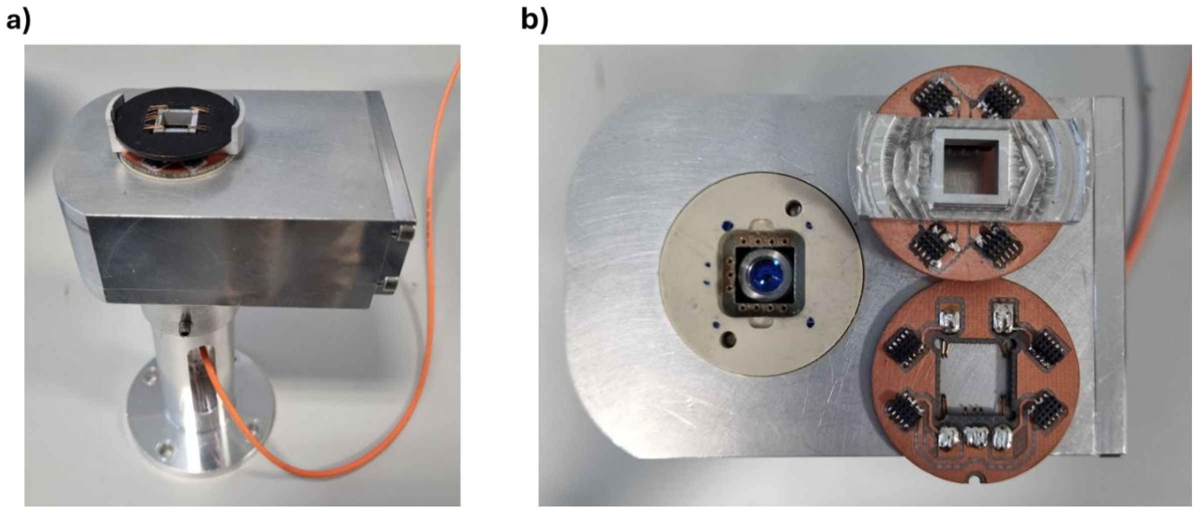

Figure S4: a) Side view of the sample stage used for operando GIWAXS measurements. The optical fibre is the orange cable. b) Top view of the sample stage. The collimator lens attached at the end of the fibre is visible in the centre of the hole.

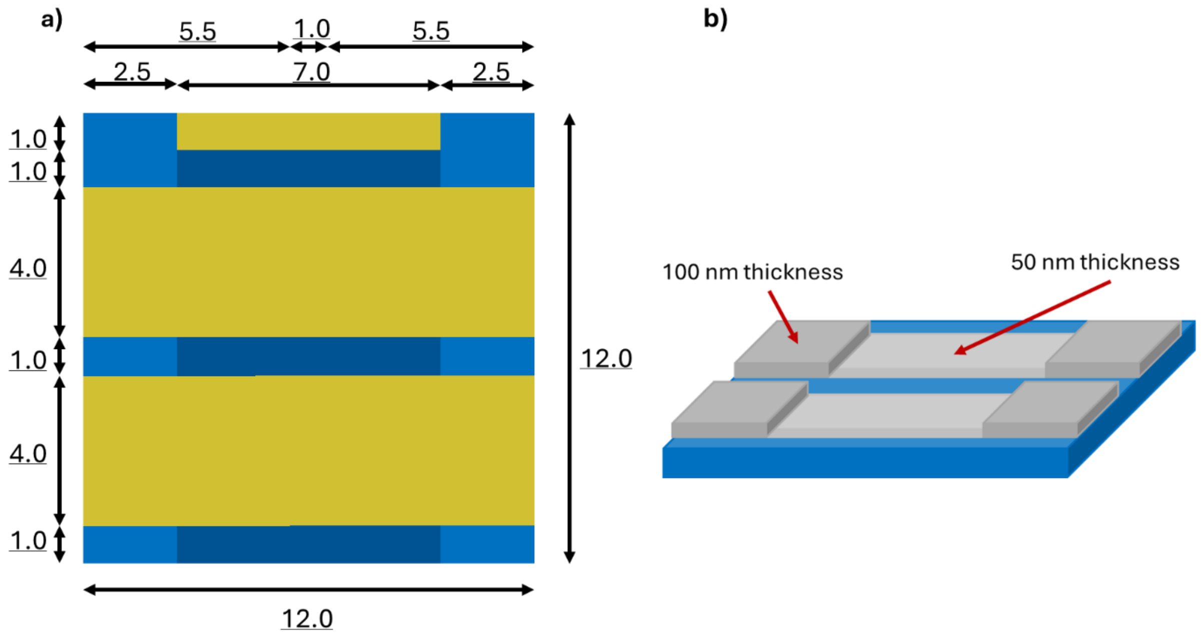

Figure S5: a) Layout mask of the GIWAXS pixels. All values are in mm. The yellow areas denote where the metal is deposited on the substrate. b) Diagram of the pixel thickness used for the GIWAXS measurements.

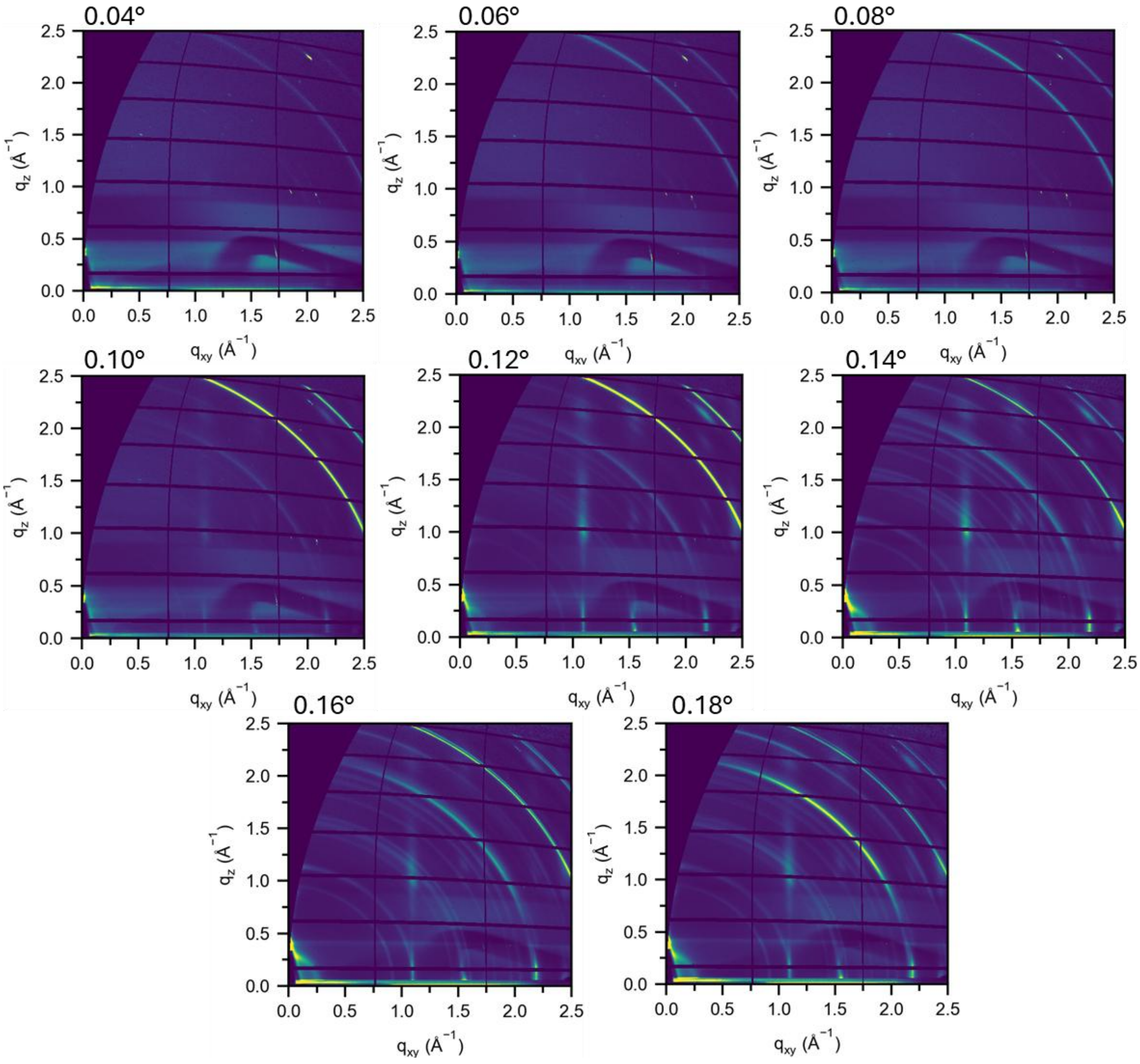

Figure S6: Series of GIWAXS images starting from 0.04° up to 0.18° taken on the pixel before applied bias.

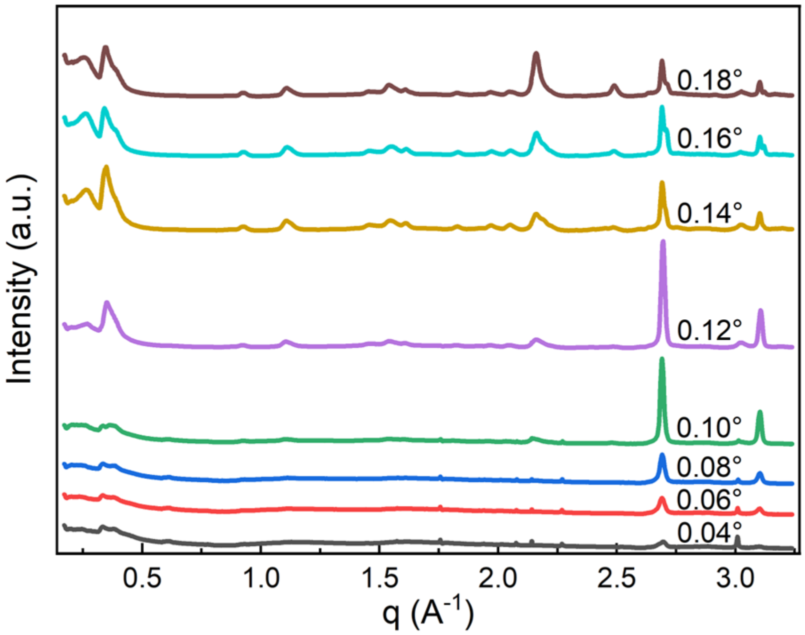

Figure S7: Corresponding integrated 1D profiles of the GIWAXS images of Figure S6.

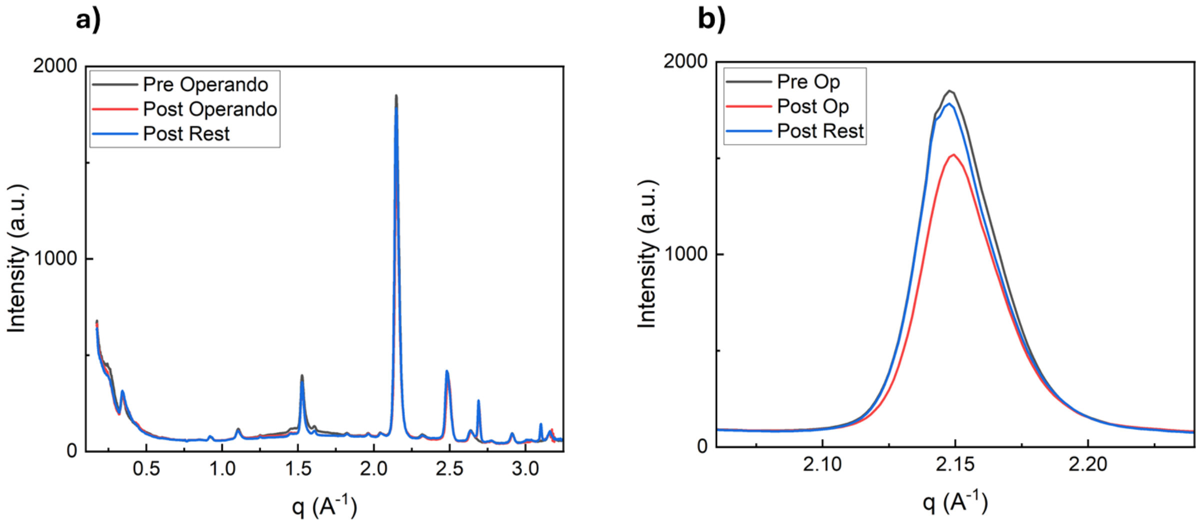

Figure S8: a) 1D line integration of the GIWAXS scans taken at 0.4° pitch angle before (black) and after (red) applied bias and after rest (blue) where no x-ray nor applied bias for 10 minutes. b) Highlight of the ITO peak at 2.15 A^{-1} which is used to ensure there is no drift between scans.

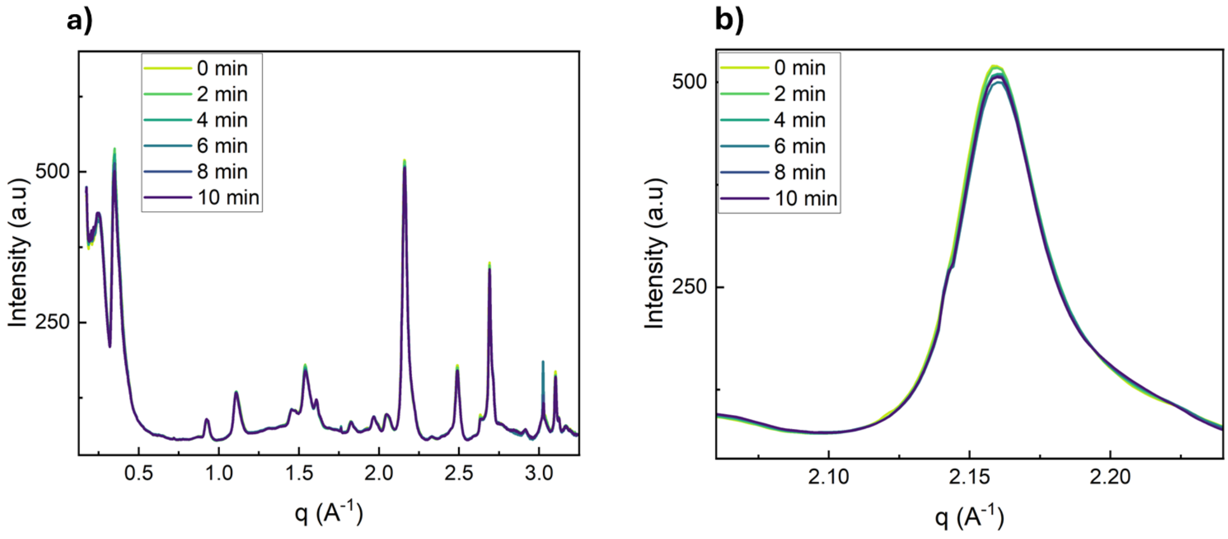

Figure S9: a) 1D line integration of the operando GIWAXS scans peaks going from 0 minutes (light green) to 10 minutes (dark blue) taken at 0.18° pitch angle. b) Highlight of the 2.15 A^{-1} ITO peak during operando measurement which is used to properly check the relevance of any possible shift in the perovskite peaks.

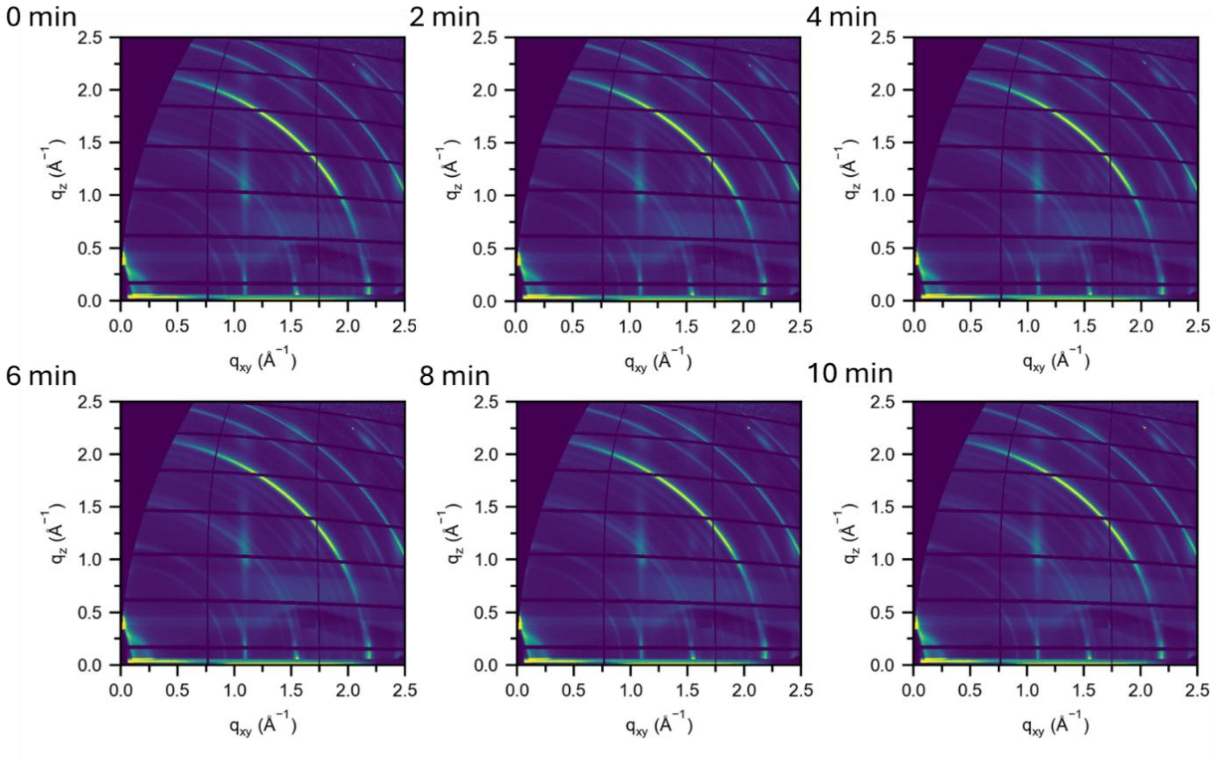

Figure S10: Corresponding GIWAXS patterns of the 1D line integrations from Figure 2g.

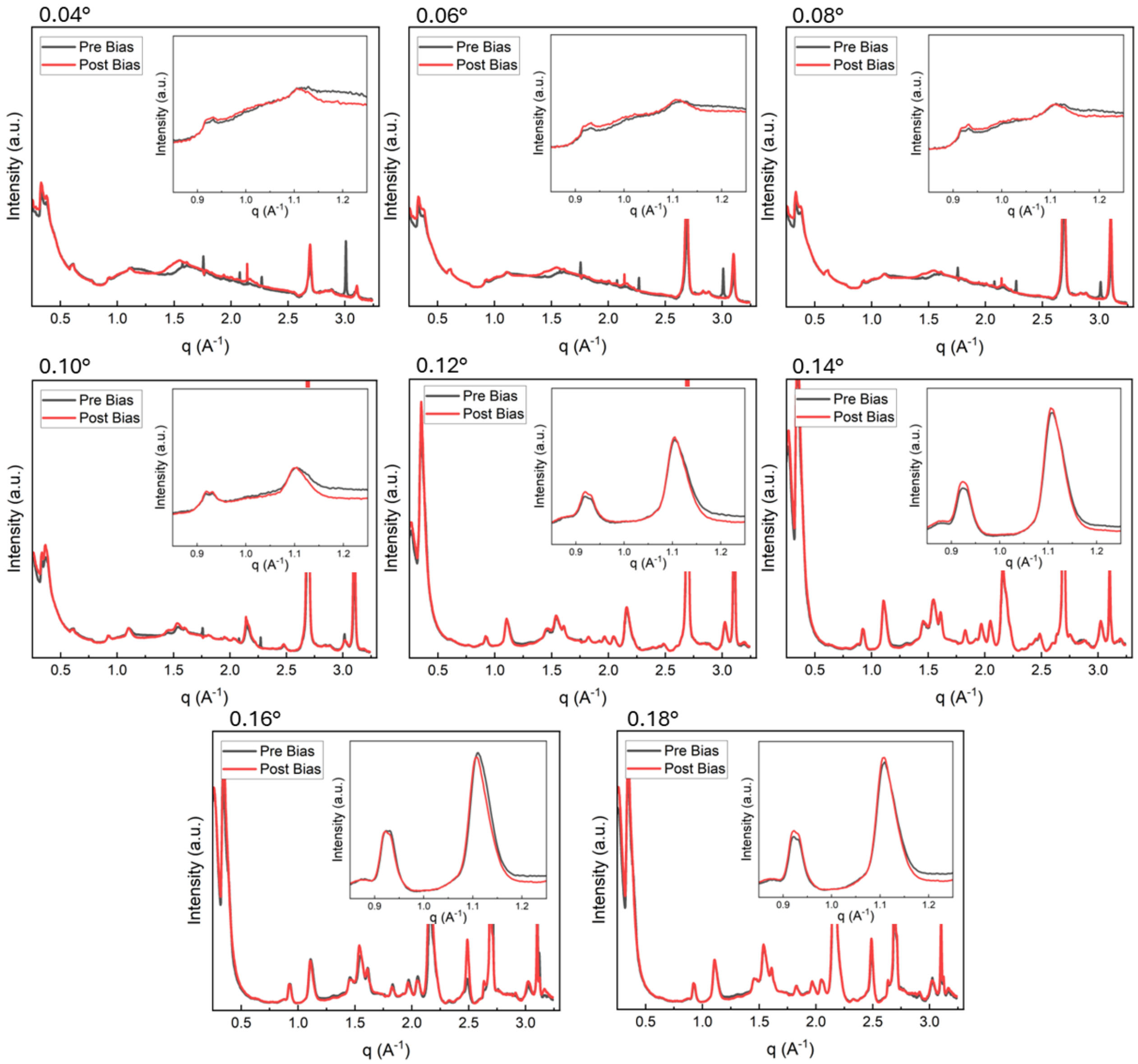

Figure S11: Comparison of the diffraction patterns of the PeLED taken on the pixel before and after electrical bias at different pitch angles starting from 0.04°. The pixels were operated for 10 minutes at 4.5 V. Inset: highlight of the main perovskite peaks.

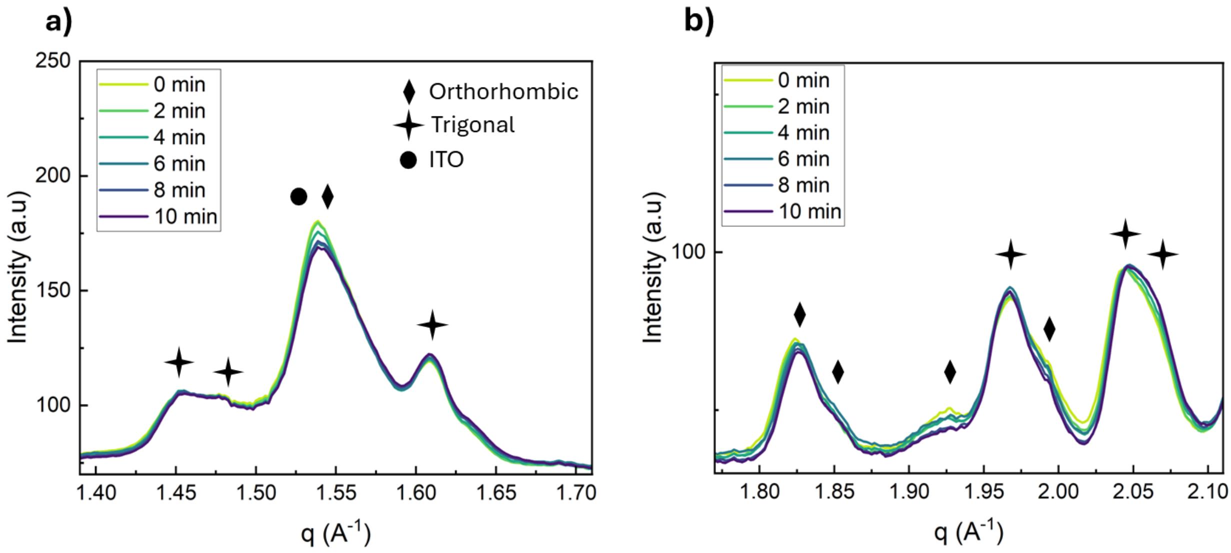

Figure S12: a-b) Highlights of Figure S 9a focusing on other perovskite peaks at higher *q*-values where we can observe the similar intensity signal trend between trigonal and orthorhombic phases.

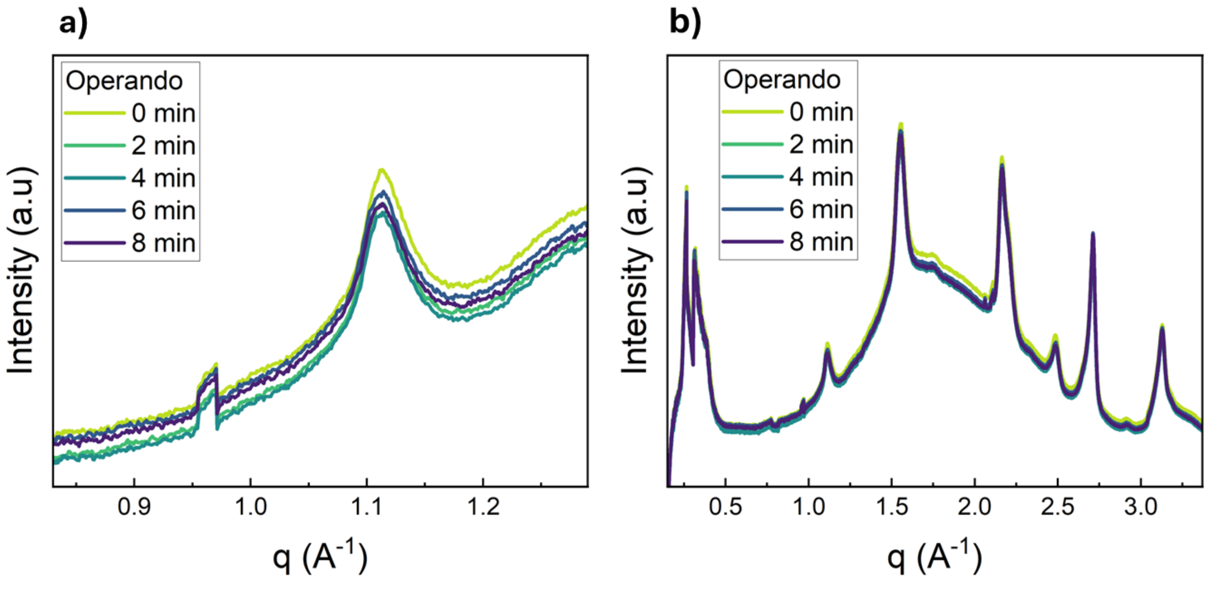

Figure S13: a) 1D integration of operando diffraction patterns of a PeLED with active perovskite layer made with precursors PEABr, CsBr, $PbCl_2$ and $PbBr_2$, with nominal ratios of 0.8:1:0.63:0.32 respectively, highlighting the perovskite peak at q = 1.11 Å^{-1}. b) Entire 1D integration of the same operando diffraction patterns.

HAXPES

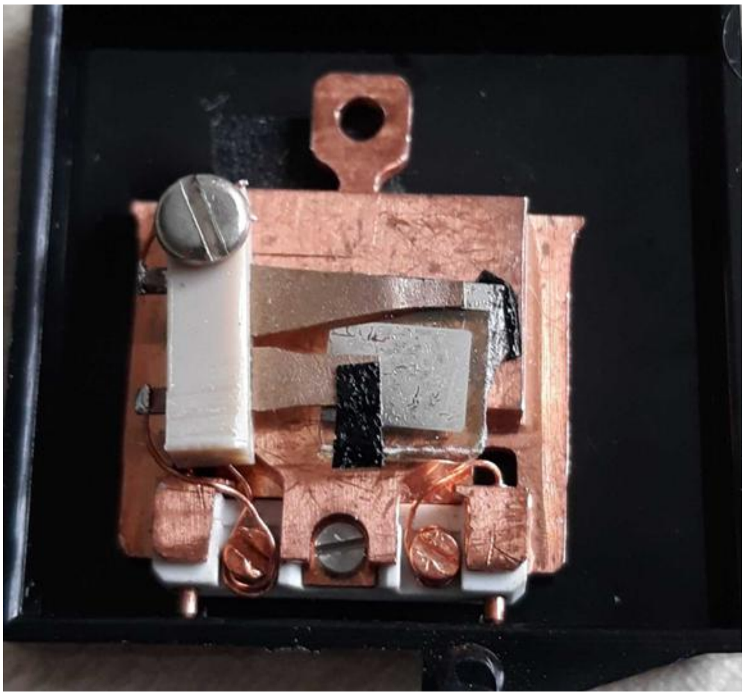

Figure S14: a) Photograph of the HAXPES samples holder with a PeLED mounted on it.

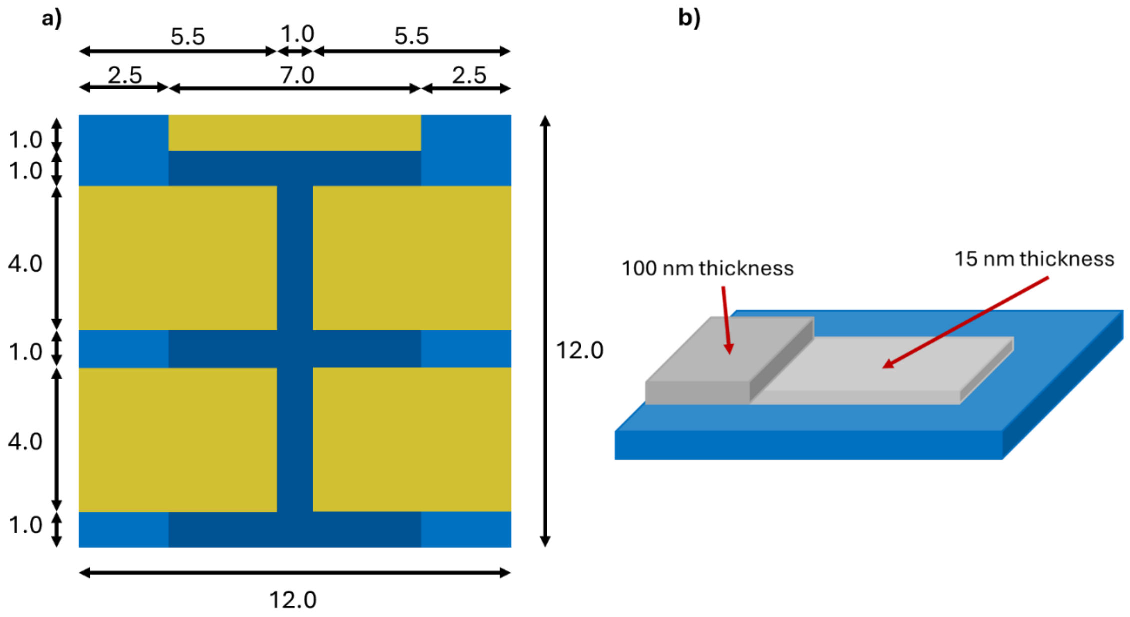

Figure S15: a) Layout of the HAXPES pixels. All values are in mm. The yellow areas denote where the metal is deposited on the substrate. b) Diagram of the pixel thickness used for the HAXPES measurements. From 1 substrate we obtained 4 pixels but only one could fit on the HAXPES holder. The glass substrates were cut into 4 with a Diamond pen after the final metal evaporation.

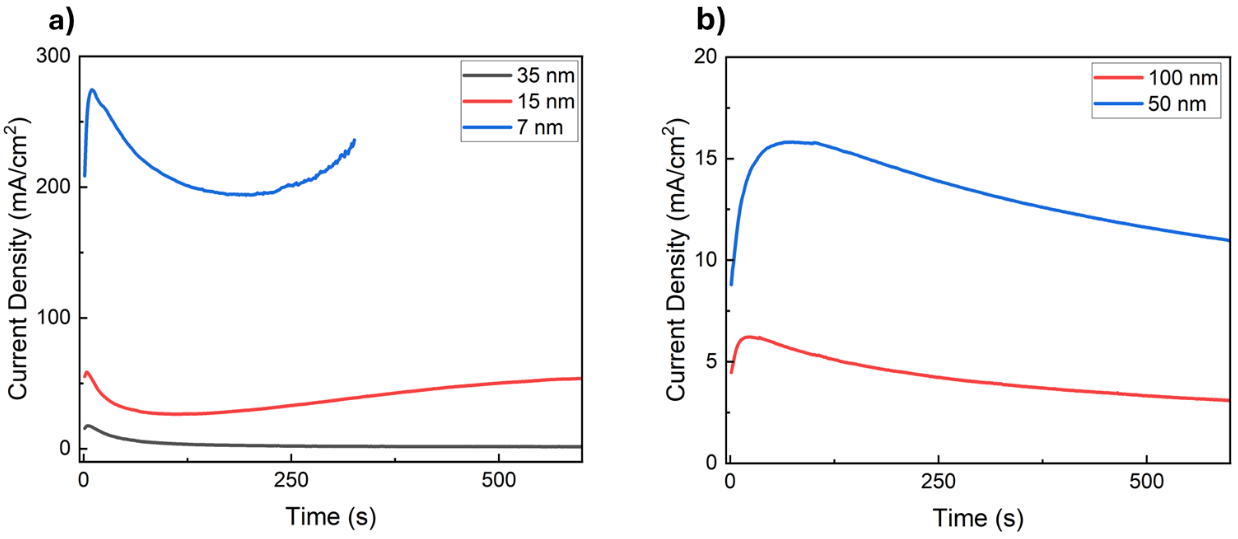

Figure S16: a) Current density over time of a series of PeLEDs with GIWAXS layout and varying thicknesses of TPBi. The PeLED with 7 nm of ETL (blue) broke down after 5 mins. PeLEDs were biased all at same 6 V for 10 mins and measured at the I07 synchrotron beamline. b) Current density over time of two PeLEDs with same 35 nm thickness of TPBi but varying aluminium thickness over the active emitting area. PeLEDs were biased all at 6 V for 10 mins and measured in house.

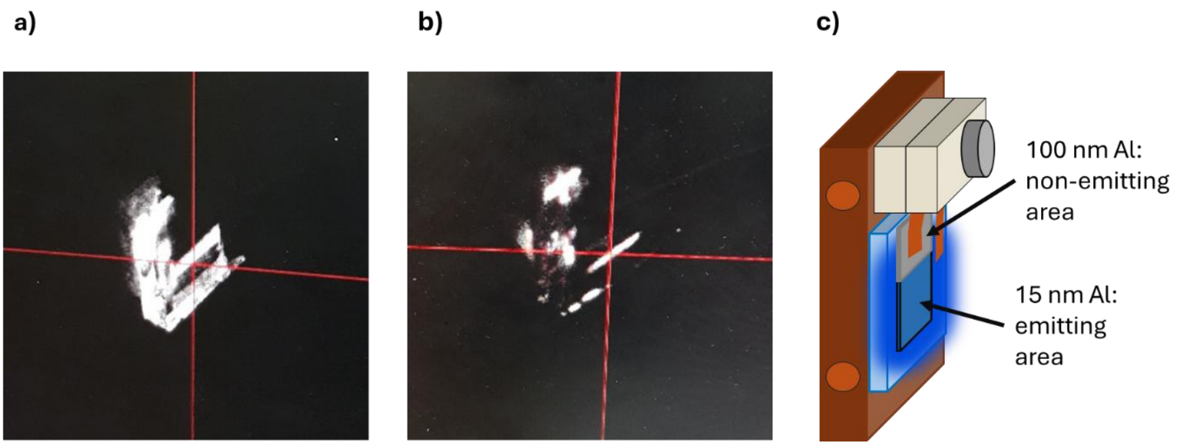

Figure S17: a) Photograph from the camera feed focused on the sample inside the UHV HAXPES chamber of the PeLED emitting light when biased during HAXPES measurements. b) Photograph of the PeLED shining from emitted X-ray induced luminescence. c) Schematic of the PeLED explaining the orientation during the HAXPES experiment. The cross hair in a) and b) indicates the X-ray beam footprint. The black line in a) in the cross hair is due to the damage left on the glass substrate by the X-rays, while the black strip above it, is the section of the electrode that was 100 nm thick. The EL is also reflected off the back of the sample stage. The substrate dimensions are 6x6 mm.

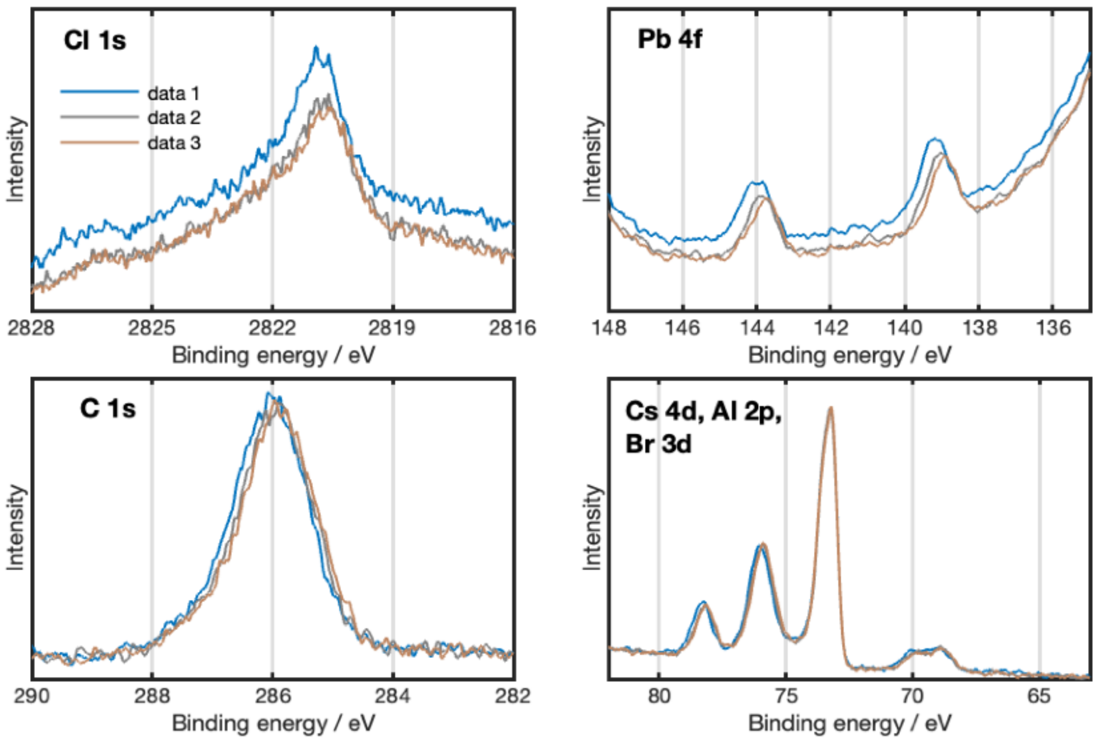

Figure S18: Representative raw core level spectra (Cl 1s, Pb 4f, C 1s, Cs 4d, Al 2p, Br 3d) on 464 LED before biasing. All spectra were energy calibrated against Fermi level on an Au foil mounted on the manipulator. No intensity normalization or background subtraction was performed.

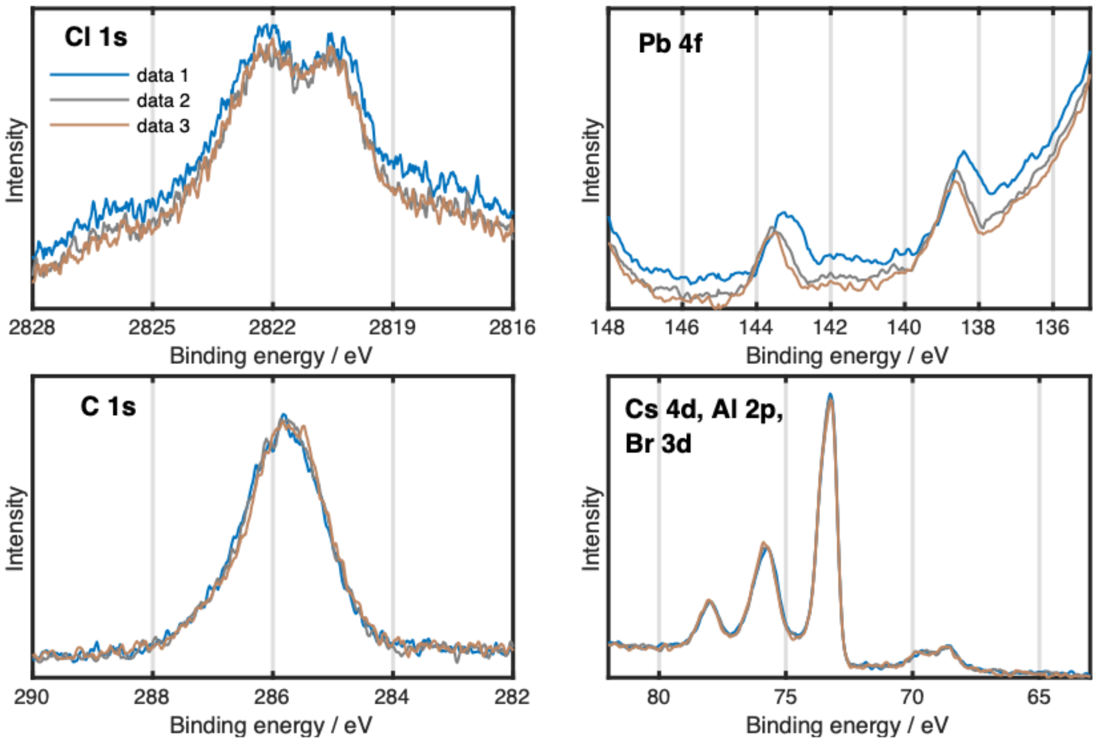

Figure S19: Representative raw core level spectra (Cl 1s, Pb 4f, C 1s, Cs 4d, Al 2p, Br 3d) on LED sample after biasing 20 minutes (without X-ray exposure). All spectra were energy calibrated against Fermi level on an Au foil mounted on the manipulator. No intensity normalization or background subtraction was performed.

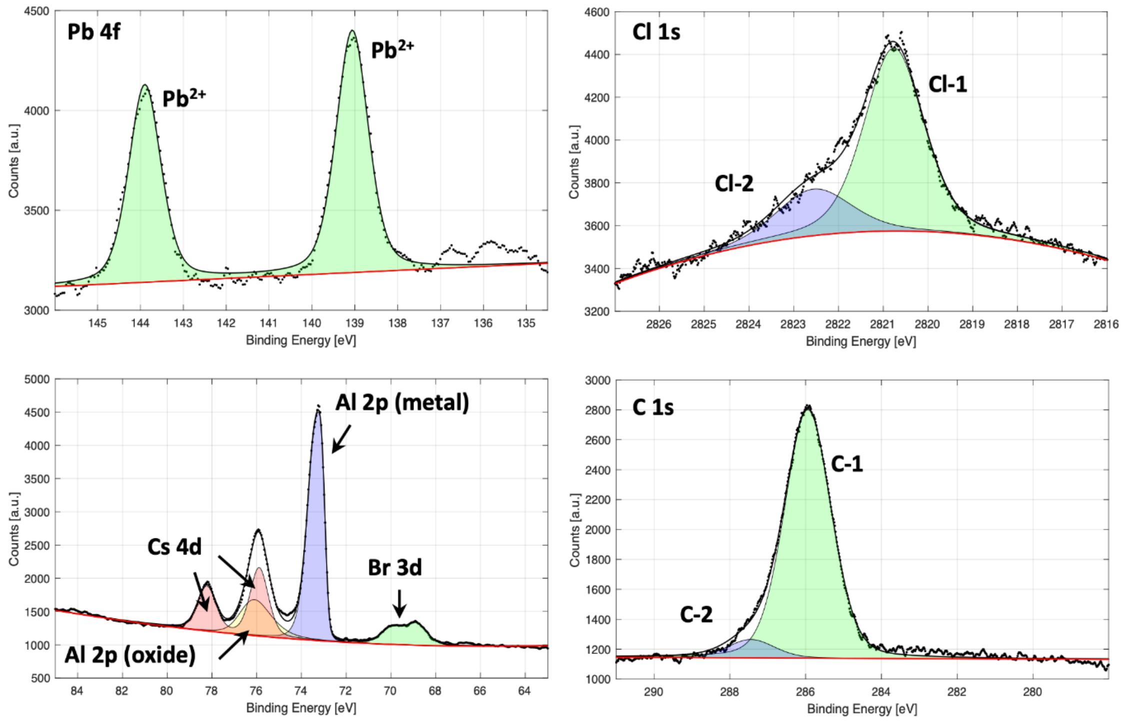

Figure S20: Fits of core level spectra (Cl 1s, Pb 4f, C 1s, 4d, Al 2p, Br 3d) on control PeLED before biasing, recorded at photon energy 6.6 keV. The different components are marked in each spectrum. Aluminium background subtraction was performed on Pb 4f and Cl 1s core level.

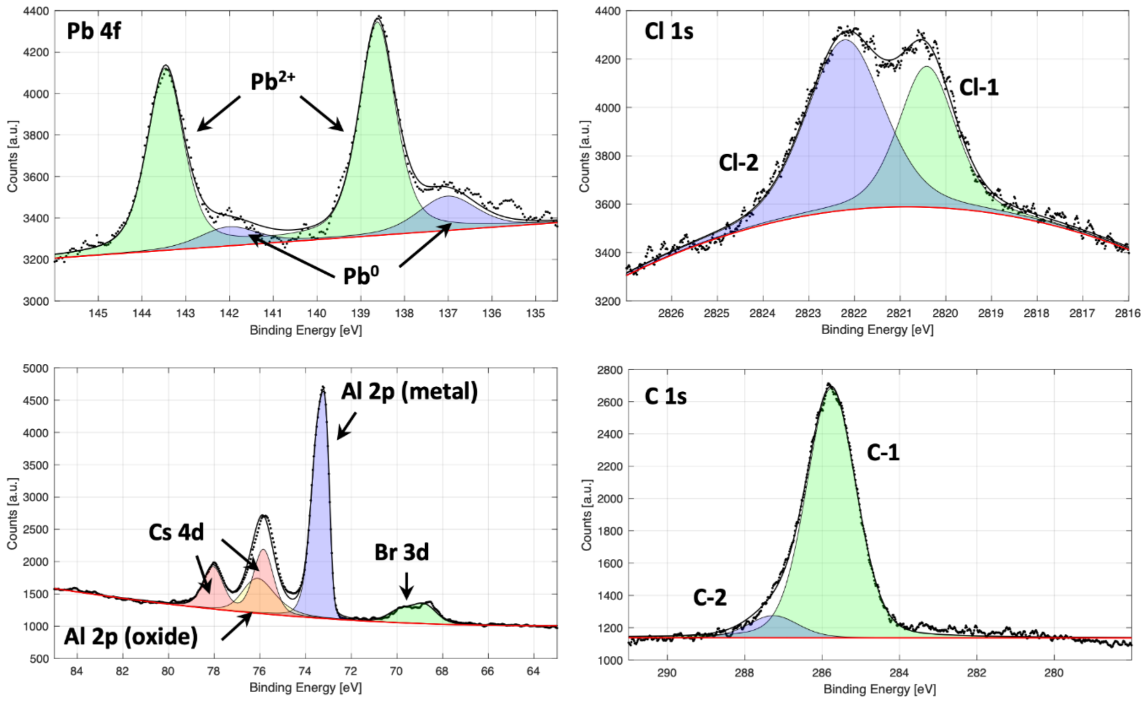

Figure S21: Fits of core level spectra (Cl 1s, Pb 4f, C 1s, Cs 4d, Al 2p, Br 3d) on control PeLED after biasing for 20 minutes, recorded at photon energy of 6.6 keV. The different components are marked in each

spectrum. Aluminium background subtraction was performed on Pb 4f and Cl 1s core level.

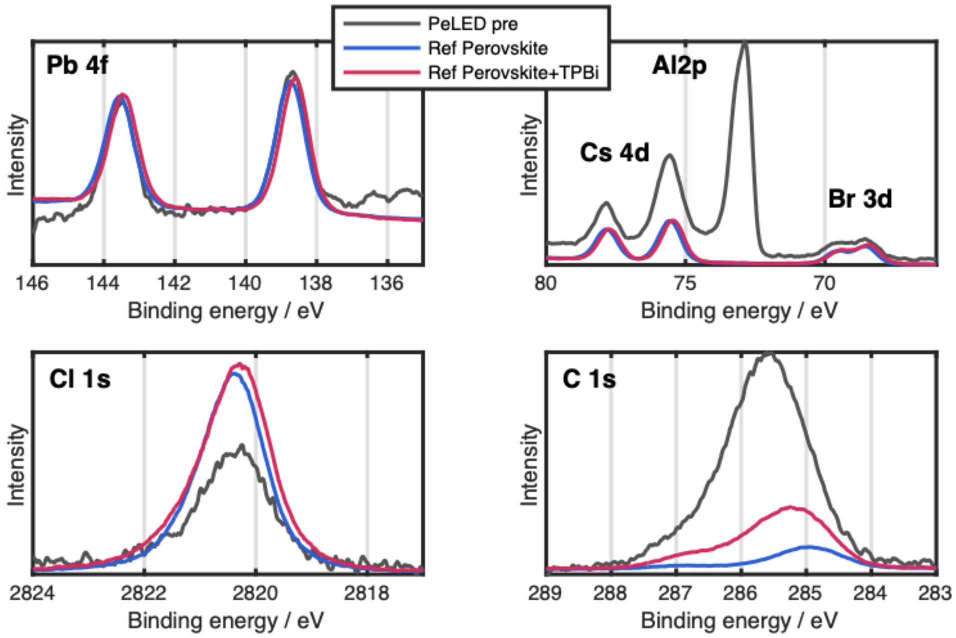

Figure S22: Core level spectra (Br 3d, Cs 4d, Pb 4f, C 1s, Cl 1s) on the PeLED before biasing (PeLED pre, black) and reference samples of the perovskite layer (Ref Perovskite, blue) and the perovskite layer with TPBi on top (Ref Perovskite + TPBi, pink), recorded at 6.6 keV with the same settings used for the PeLEDs. The PeLED pre spectra are energy calibrated to Pb 4f of the reference samples. All reference spectra are energy calibrated against Au $4f_{7/2}$. All spectra are intensity normalized towards the Pb 4f.

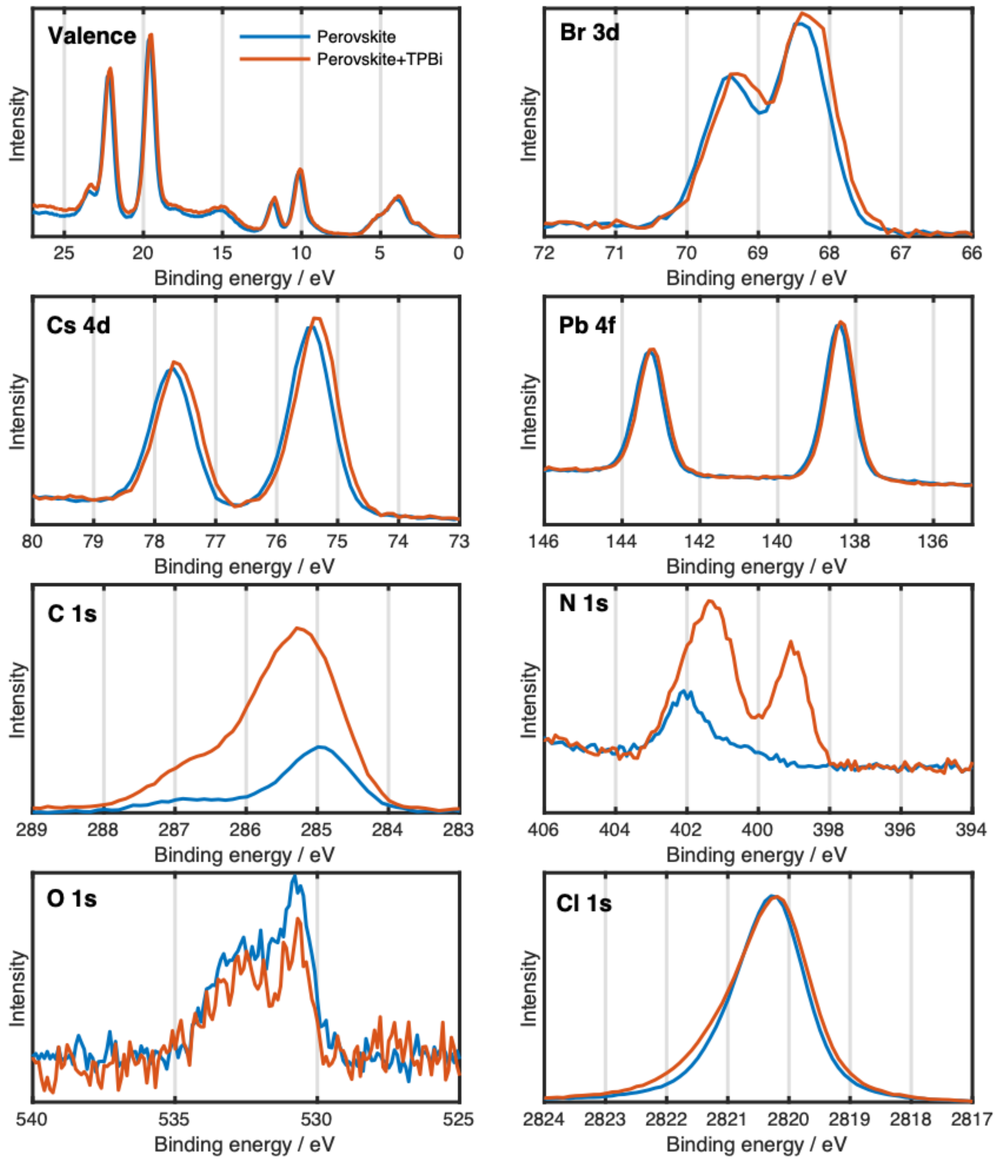

Figure S23: Core level spectra (Br 3d, Cs 4d, Pb 4f, C 1s, N 1s, O 1s, Cl 1s) and Valence region on reference samples of the perovskite layer (blue) and the perovskite layer with TPBi on top (orange), recorded at 6.6 keV. All spectra are energy calibrated against Au $4f_{7/2}$ and intensity normalized towards the Pb 4f.

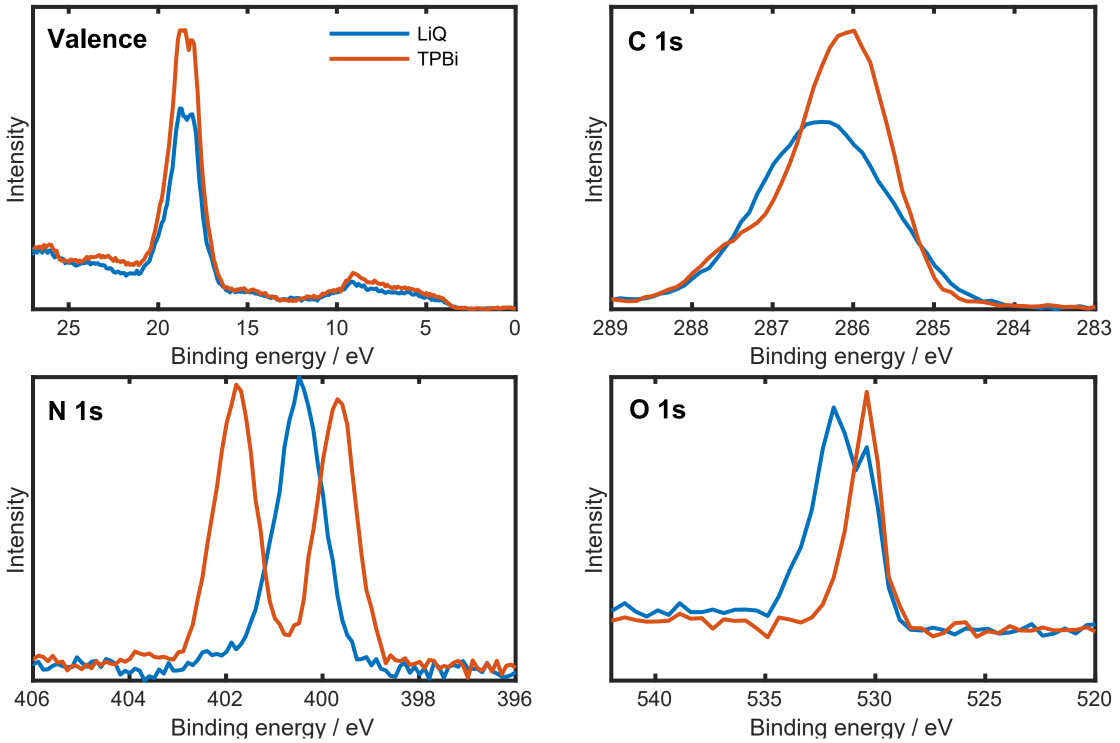

Figure S24: Core level spectra (C 1s, N 1s, O 1s) and Valence region on reference samples of LiQ (blue) and TPBi (orange), recorded at 6.6 keV. The O 1s spectrum is taken from a survey, also recorded at 6.6 keV. All spectra are energy calibrated against Au $4f_{7/2}$. No intensity normalization was performed.

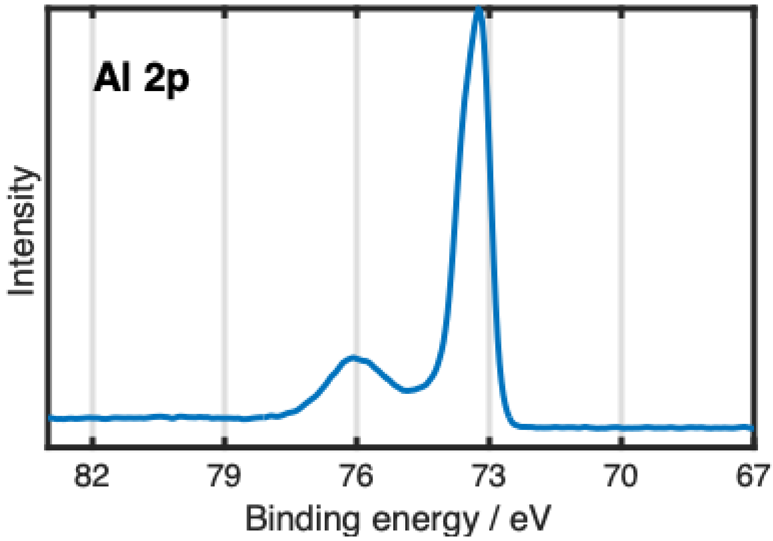

Figure S25: Al 2p core level spectrum recorded on thick aluminium electrode on PeLED at 6.6 keV. The spectrum is energy calibrated against Au $4f_{7/2}$. No intensity normalization was performed.

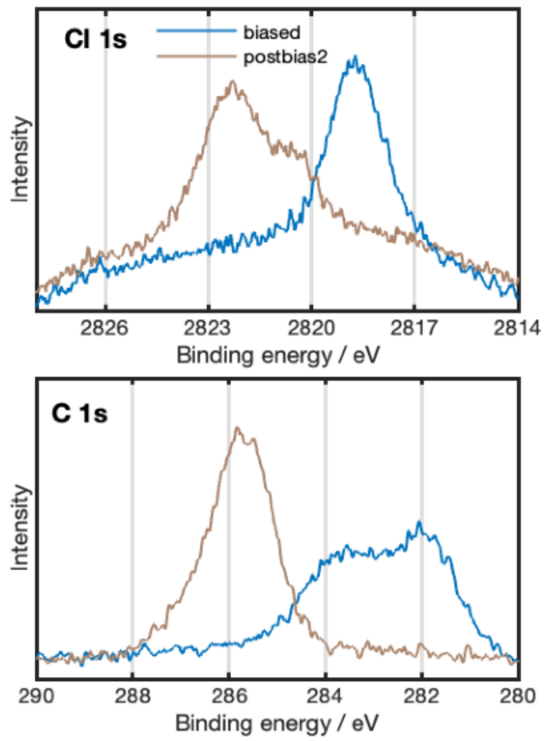

Figure S26: Representative raw core level spectra (Cl 1s, Pb 4f, C 1s, Cs 4d, Al 2p, Br 3d) on the PeLED during and after biasing. All spectra are energy calibrated against Fermi level on an Au foil mounted on the manipulator. No background subtraction or intensity normalization was performed.

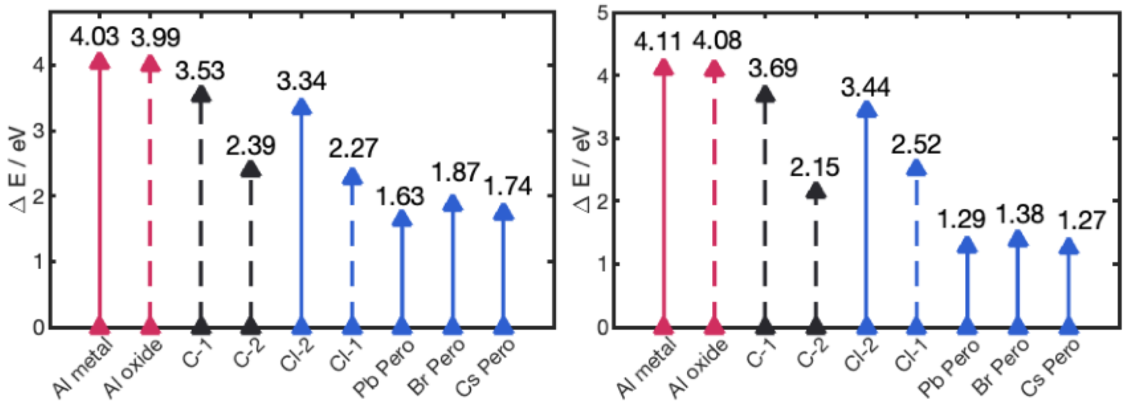

Figure S27: Binding energy shifts during biasing at 4.5 V of repeat samples 1 (left) and 2 (right).

HAXPES on PeLEDs with varying TPBi thickness

Figure S28 shows core level spectra (Cs 4d, Al 2p, Br 3d, Pb 4f, C 1s, N 1s, O 1s, Cl 2s, Br 3s) of PeLED reference samples with varying TPBi thicknesses without biasing, recorded at 6.6 keV, energy calibrated against Pb $4f_{7/2}$ and intensity normalized to Al 2p (metal). The thicknesses are an estimation and may be somewhat lower than expected due to evaporator difficulties. Significant differences can be observed for different TPBi thicknesses. The Pb 4f core level spectra show no Pb^0 formation for the sample with thickest TPBi layer, while all other samples show substantial Pb^0. Notably, the PeLED without TPBi shows similar amounts of Pb^{2+} and Pb^0, while the 7 nm TPBi PeLED shows almost no Pb^{2+}. Furthermore, thinner (or no) TPBi layers show substantial increase in Cl 2s. Br 3s and Br 3d intensities were lowest for the 30 nm TPBi PeLED, while the other PeLEDs showed similar amounts.

The Al 2p metal peak shifts to higher binding energy with increasing TPBi thickness relative to the Pb 4f peak. Both Cs 4d and Br 3d show significant increase of new species at higher binding energy in the samples with 7 nm TPBi.

The C 1s and N 1s core level spectra confirm the presence of TPBi in the PeLEDs with TPBi. Notably, a shift to higher binding energy is visible in the C 1s spectrum for the 30 nm TPBi PeLED, and the PeLED without TPBi also shows some non-TPBi-related C 1s features. Lastly, decreasing O 1s content can be observed with increasing TPBi thickness.

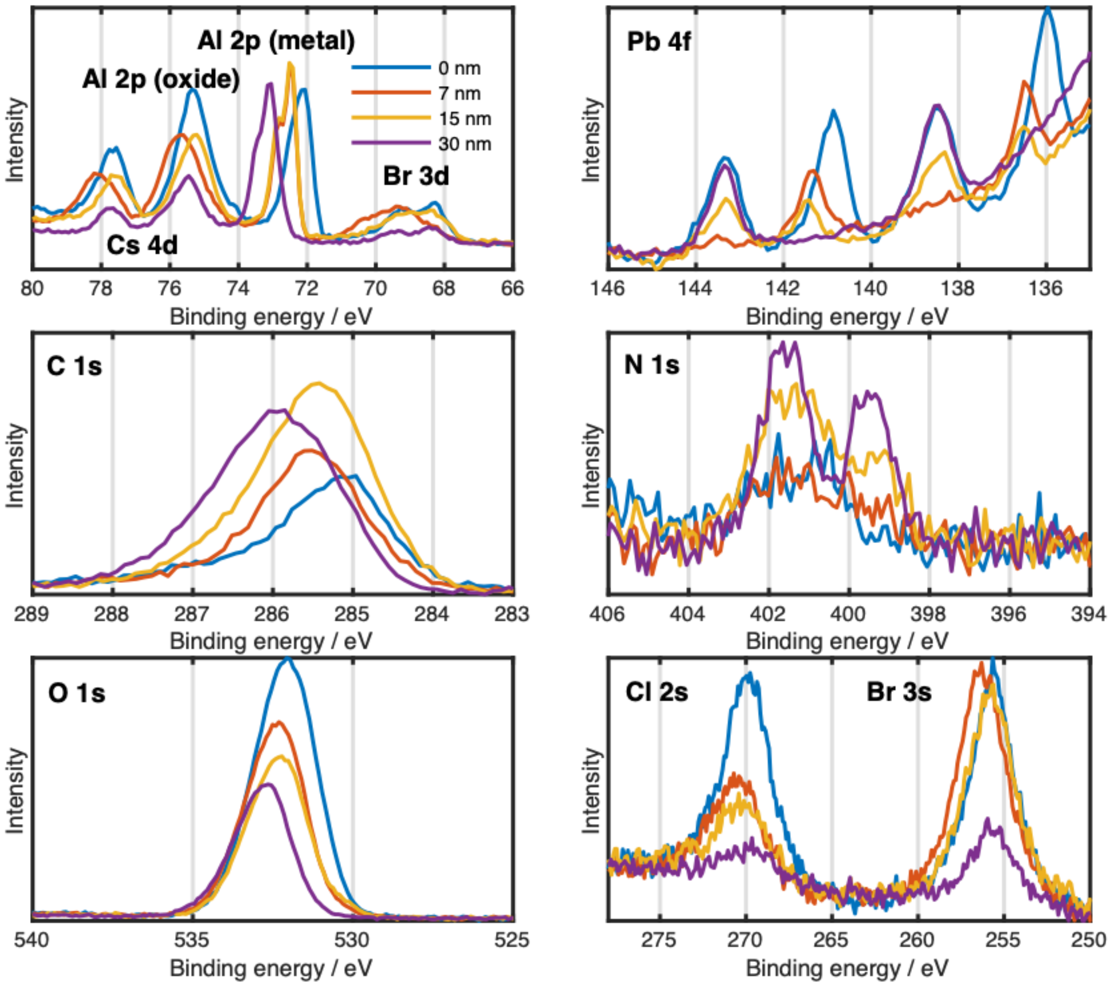

Figure S28: Core level spectra (Br 3d, Al 2p, Cs 4d, Pb 4f, C 1s, N 1s, O 1s, Cl 2s, Br 3s) on full LEDs with varying TPBi thicknesses: 0 nm (blue), 7 nm (orange), 15 nm (yellow) and 35 nm (purple). No bias was applied on these PeLEDs. All spectra were recorded at 6.6 keV, energy calibrated against Pb $4f_{7/2}$ and intensity normalized towards Al 2p (metal). The Al thickness on the measured area was 15 nm.

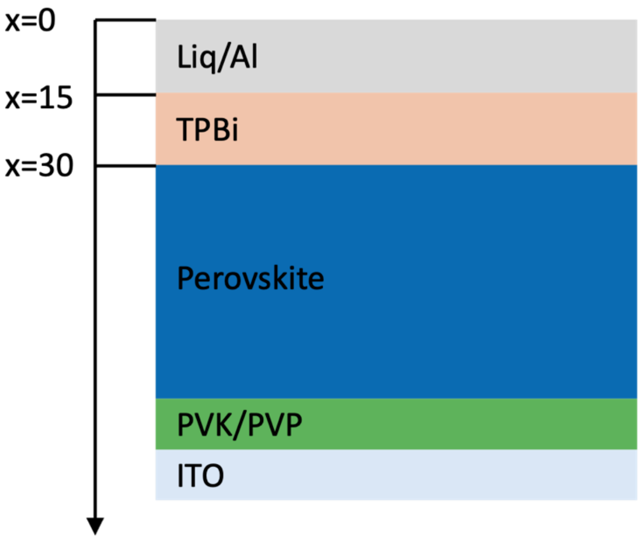

Figure S29: Schematic of the PeLED structure used to calculate the relative intensities (I), where *x* is the distance in nm from the surface of the PeLED. Due to the inelastic mean free path (λ) of photoelectrons, species closer to the surface will be detected more than species further in the bulk. For Cl 1s we estimated λ to be 6.9 nm (Table S2). In a simple calculation using the equations below and setting the integration boundaries corresponding to the respective layers, we can estimate relative intensities (I) for a Cl in the TPBi layer and in the perovskite layer. For simplicity, this calculation is based on an even distribution of Cl in the TPBi layer and in the perovskite. From these calculations, we can estimate that on average Cl from the TPBi layer is detected 7.8 times more efficiently than Cl from the perovskite layer.

$$I_{Cl\ (TPBi)} \propto \int_{15}^{30} e^{-\frac{x}{\lambda}} dx \cong 0.7$$

$$I_{Cl\ (Perovskite)} \propto \int_{30}^{\infty} e^{-\frac{x}{\lambda}} dx \cong 0.09$$

$$\frac{I_{Cl\ (TPBi)}}{I_{Cl\ (Perovskite)}} = \frac{0.7}{0.09} \cong 7.8$$

Comparison with pure Br PeLEDs

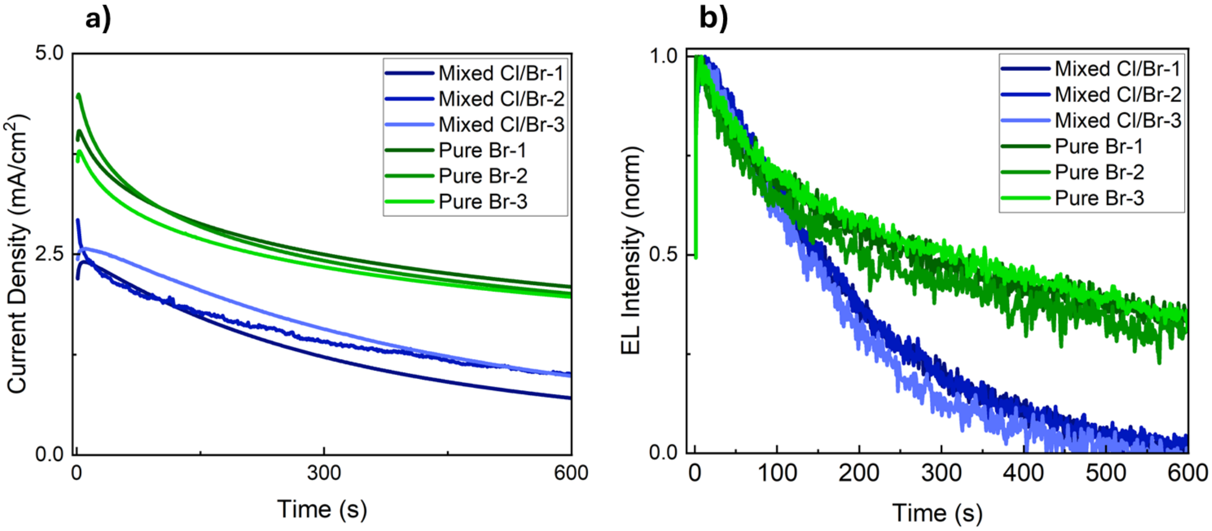

Figure S30: Comparison of the current density (a) and normalized EL intensity (b) between deep blue and green PeLEDs whilst biasing at 4.5 V for 10 minutes. The PeLEDs were obtained starting with the formula p-FPEABr, CsBr, FABr, and ($PbCl_2$ + $PbBr_2$). Deep blue was achieved with the ratio 2:1 between $PbCl_2$/$PbBr_2$ and the green emission was obtained by employing only $PbBr_2$. The ratio between the cations p-FPEABr and CsBr was kept the same to exclude any 2D/3D contribution. We note that the overall device architectures, compositions, additives and processing have been optimised for the deep blue systems, and thus the pure Br device is not optimised in this configuration but is used as a means to demonstrate the critical role of Cl in the degradation pathway through an otherwise like-for-like comparison.

References

(1) Anaya, M.; Rand, B. P.; Holmes, R. J.; Credgington, D.; Bolink, H. J.; Friend, R. H.; Wang, J.; Greenham, N. C.; Stranks, S. D. Best Practices for Measuring Emerging Light-Emitting Diode Technologies. *Nat Photon*. **2019**, *13*, 818–821. https://doi.org/10.1038/s41566-019-0543-y.

(2) Ruggeri, E.; Anaya, M.; Gałkowski, K.; Abfalterer, A.; Chiang, Y.-H.; Ji, K.; Andaji-Garmaroudi, Z.; Stranks, S. D.; Ruggeri, E.; Anaya, M.; Gałkowski, K.; Abfalterer, A.; Chiang, Y.-H.; Ji, K.; Andaji-Garmaroudi, Z.; Stranks, S. D. Halide Remixing under Device Operation Imparts Stability on Mixed-Cation Mixed-Halide Perovskite Solar Cells. *Advanced Materials* **2022**, *34* (36), 2202163. https://doi.org/10.1002/ADMA.202202163.

(3) Lee, T. L.; Duncan, D. A. A Two-Color Beamline for Electron Spectroscopies at Diamond Light Source. *Synchrotron Radiat News* **2018**, *31* (4), 16–22. https://doi.org/10.1080/08940886.2018.1483653.

(4) Shirley, D. A. High-Resolution X-Ray Photoemission Spectrum of the Valence Bands of Gold. *Phys Rev B* **1972**, *5* (12), 4709. https://doi.org/10.1103/PhysRevB.5.4709.

(5) Powell, C. J. Practical Guide for Inelastic Mean Free Paths, Effective Attenuation Lengths, Mean Escape Depths, and Information Depths in x-Ray Photoelectron Spectroscopy. *Journal of Vacuum Science & Technology A: Vacuum, Surfaces, and Films* **2020**, *38* (2). https://doi.org/10.1116/1.5141079.
